# Quantum expander for gravitational-wave observatories

**DOI:** 10.1038/s41377-019-0230-2

**Published:** 2019-12-11

**Authors:** Mikhail Korobko, Yiqiu Ma, Yanbei Chen, Roman Schnabel

**Affiliations:** 1 Institut für Laserphysik und Zentrum für Optische Quantentechnologien, Universität Hamburg, Luruper Chaussee 149, 22761 Hamburg, Germany; 2 Theoretical Astrophysics 350-17, California Institute of Technology, Pasadena, CA 91125 USA

**Keywords:** Nonlinear optics, Quantum optics, Optical metrology

## Abstract

The quantum uncertainty of laser light limits the sensitivity of gravitational-wave observatories. Over the past 30 years, techniques for squeezing the quantum uncertainty, as well as for enhancing gravitational-wave signals with optical resonators have been invented. Resonators, however, have finite linewidths, and the high signal frequencies that are produced during the highly scientifically interesting ring-down of astrophysical compact-binary mergers still cannot be resolved. Here, we propose a purely optical approach for expanding the detection bandwidth. It uses quantum uncertainty squeezing inside one of the optical resonators, compensating for the finite resonators’ linewidths while keeping the low-frequency sensitivity unchanged. This quantum expander is intended to enhance the sensitivity of future gravitational-wave detectors, and we suggest the use of this new tool in other cavity-enhanced metrological experiments.

## Gravitational waves: squeezing out uncertainty for better detection

Gravitational waves produced by cataclysmic events in the universe could be detected with greater sensitivity using an innovative optical procedure to reduce the uncertainties that quantum effects impose on existing laser-based methods. Gravitational waves can be detected by monitoring the fluctuating disturbances they cause in the path length of laser light. Mikhail Korobko and colleagues at the University of Hamburg, Germany, with co-workers at California Institute of Technology, USA, propose what they call a “quantum expander” process to increase the detector sensitivity. The predicted improvement is based on the interaction between a crystal placed within the detector and the light inside cavities that is perturbed by a gravitational wave. The researchers describe their procedure as expanding the range over which quantum mechanical uncertainties are squeezed out. The innovation may also be applicable in other measurement devices based on light moving within cavities.

## Introduction

The dawn of gravitational-wave (GW) astronomy began with the historic detection of binary black hole coalescence in 2015^1^ and several more detection events that followed in subsequent years^2^. The latest observation of gravitational waves was from a binary neutron star inspiral. It was succeeded by observations of a broad spectrum of electromagnetic counterparts^3,4^, demonstrating that gravitational-wave astronomy is invaluable for understanding the Universe^5,6^. Further increasing the sensitivity of gravitational-wave observatories (GWOs) is of utmost importance to maximize the scientific output of combined multi-messenger astronomical observations.

**Fig. 1:**
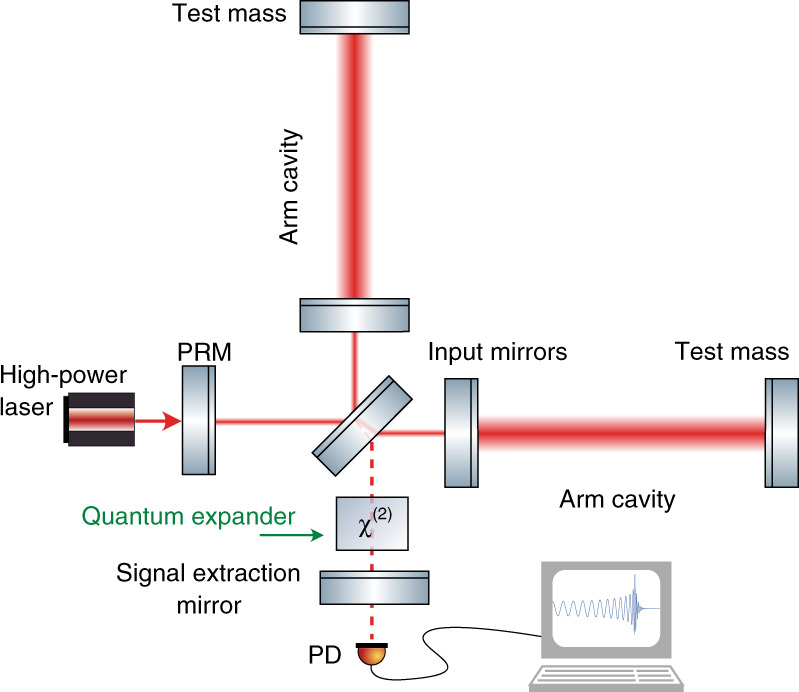
Conceptual representation of the GW observatory with our quantum expander. The relative change in the distance between the central beam splitter and the test masses due to a gravitational wave is measured on the signal port with photodiode PD. Optical cavities in the arms are used to enhance the light power and the signal. Additional mirrors independently enhance the signal (signal extraction mirror, SE mirror) and power (power recycling mirror, PRM). We add a nonlinear *χ*^(2)^ crystal into the SE cavity, formed by the SE mirror and input mirrors, which creates an internally squeezed light field to enhance the high-frequency sensitivity and expand the detection bandwidth.

Gravitational-wave observatories, such as Advanced LIGO^7^, Advanced Virgo^8^, GEO600^9^, and KAGRA^10^, are based on the Michelson interferometer topology (see Fig. 1), where the incoming gravitational wave changes the relative optical path length of two interferometer arms. The ability of the observatories to measure gravitational waves is limited by various disturbances that also change the differential path length or manifest themselves as such. The main noise source at signal frequencies above ~50 Hz in the current generation of GWOs is the quantum uncertainty of the light field, which results in shot noise (photon counting noise)^7,11^. Noise at lower frequencies has contributions of several origins, such as Brownian motion of the mirror surfaces and suspensions or quantum radiation pressure noise, which comes from mirrors’ random motion due to quantum fluctuations of light power^12,13^. All these noise sources contribute to the photocurrent of the photodiode placed on the signal port of the detector. The observatory’s sensitivity to the GW signal, i.e., its ability to discriminate between the GW signal and noise is given by the observatory’s signal-to-noise ratio (SNR). For a given set of parameters of the detector, e.g., the interferometer’s arm length or laser power, its sensitivity is ultimately limited by its “quantum Cramer-Rao bound” (QCRB)^14^. For continuous signals, the QCRB at every frequency is determined by the radiation pressure force exerted on the test mass by quantum fluctuations of the light field in a pure state^15^. The higher is this quantum radiation-pressure force on the mirror, the higher is the uncertainty in its momentum, and thus the better can the position be resolved, according to the Heisenberg uncertainty relation^16^. The QCRB represents an ultimate limit on the sensitivity of the detector, which can only be saturated in the absence of decoherence and under the optimal measurement protocol. For example, reaching this limit at low frequencies in GWDs requires implementing the back-action evasion measurement scheme, e.g., a variational readout^17^, such that the sensitivity is fully limited by shot noise.

Generally, the QCRB is decreased when the squeezed states of light are added to the interferometer configuration (such that the quantum uncertainty in the amplitude of the light field is increased)^12,18,19^. The injection of squeezed states has become a well-established technique for GWOs^20–22^.

A classical way to lower the QCRB is to increase the signal response of the detector by optical resonators, as implemented already in the first generation of GWOs^23^. The resonance buildup of optical energy in resonators increases the radiation pressure force^16^ and hence lowers the QCRB. The current (second) generation design includes Fabry-Perot cavities in the arms and a signal-extraction (SE) cavity on the dark port of the detector^24^. Resonators, however, significantly lower the QCRB only at frequencies below the resonator’s linewidth, i.e., they *reduce* the observatory’s detection bandwidth^25^. The injection of squeezed states, mentioned above, is not able to counteract the loss of bandwidth due to resonators.

The issue of detector *bandwidth* has become crucial in the era of multi-messenger astronomy^3^. Information about the physics of extremal nuclear matter is hidden in the waveforms of gravitational waves radiated from the post-merger remnants of binary neutron star systems^26^. Obtaining this information is important for unraveling the physics of compact astrophysical objects—the engines that drive gamma-ray bursts, the origin of heavy elements and possible modifications to general relativity^27,28^. These waveforms have typical frequencies above 1 kHz, where the sensitivity of current observatories degrades due to limited bandwidth.

Over the past 20 years, the challenge of increasing the bandwidth without changing the peak sensitivity at low frequencies has become one of the cornerstones for the design of future GWOs^29,30^. Previous concepts involved unstable optomechanical or atomic systems operating in the so-called “negative dispersion” regime^31–35^.

In this work, we propose a new and all-optical concept without instabilities that targets the same goal, i.e., arbitrary expansion of the detection bandwidth, given a sufficiently low quantum decoherence. This quantum-expanded signal extraction concept is based on the optical parametric amplification process *inside* the interferometer, which allows an increase in the quantum fluctuations in the amplitude of the light by introducing quantum correlations, thereby providing a new knob to turn for reducing the QCRB. Due to the optical coupling between the cavities, the quantum uncertainty at high frequencies gets squeezed such that it compensates for the reduction in signal enhancement due to the cavity linewidth. At low frequencies, neither the signal nor the quantum noise changes, which maintains the existing sensitivity in an optimized state for observing the pre-merger stages of binary coalescence. Our approach is fully compatible with other enhancements to the detector design, such as injection of frequency-dependent squeezed light and variational readout^17,36–38^.

Placing an optical parametric amplifier inside the detector has been considered for other purposes before, i.e., for increasing the low-frequency^39^ or mid-frequency^40–42^ sensitivity, yet all-optical quantum expansion of bandwidth has never been proposed so far.

## Results

### Hamiltonian of the quantum expander

In the future, GW interferometers will operate with the signal port at the dark fringe. In this operating condition, all of the light power pumped into the interferometer is reflected towards the laser source. The only light that leaves the interferometer through the dark signal port corresponds to the signal caused by the dynamic change in the differential arm length, e.g., due to a GW. The zero-point fluctuation that enters the dark signal port defines the shot noise of the interferometer.

**Fig. 2:**
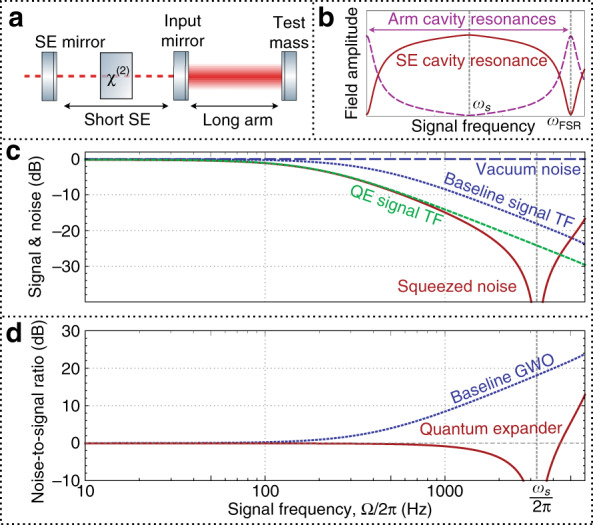
Concept of the quantum expander. **a** Model system of two coupled cavities, arm and signal extraction, with a nonlinear crystal inside the SE cavity; **b** resonance enhancement of the SE mode (solid red) at frequencies close to *ω*_*s*_ and suppression at low frequencies, with two longitudinal resonances of the arm cavity (dashed black) separated by a free spectral range (*ω*_FSR_); **c** suppression of the shot noise at high frequency by the quantum expander (red) compared to the vacuum level (black), in comparison to the scaling of the signal transfer function (TF) due to the cavity linewidth with the quantum expander (green) and without it (black), where the signal is suppressed by 6 dB due to the parametric process; **d** noise-to-signal ratio for the detector with the quantum expander (red) and without it (black). In **c**, the quantum expander noise squeezing has exactly the same scaling as signal reduction due to the cavity bandwidth, so the bandwidth of the noise-to-signal ratio is expanded, as seen in **d**.

With respect to the quantum noise and the signal, the interferometer topology can be conceptually represented by a simpler system of two coupled cavities:^43^ the arm cavity with optical mode $\widehat a$, and the signal-extraction cavity, which is formed by the front mirror of the arm cavity and the signal-extraction mirror, with optical mode $\widehat a_q$; see Fig. 2a. The two modes are coupled through the partially reflective front mirror of the arm cavity, with a coupling frequency *ω*_*s*_, which depends on the front mirror’s reflectivity. For illustrative purposes, we limit the discussion to the interaction of these two modes, while the complete description should include the modes of the next longitudinal resonances of the arm cavity, separated by one free spectral range. In this approximation, the system can be described by the standard Hamiltonian for coupled harmonic oscillators: $\widehat H/\hbar = \omega _0\widehat a^\dagger \widehat a + \omega _0\widehat a_q^\dagger \widehat a_q + \omega _s(\widehat a_q^\dagger \widehat a + \widehat a^\dagger \widehat a_q)$. If the system is continuously excited at one of the normal frequencies, *ω*_0_ ± *ω*_*s*_, the excitation energy is equally split between the two modes $\widehat a$ and $\widehat a_q$. However, when one of the modes (e.g., $\widehat a_q$) is continuously excited at *ω*_0_, the complete energy is redistributed into the other mode (e.g., $\widehat a$). In this way, when mode $\widehat a_q$ is open to the environment and driven by the incoming zero-point fluctuation, its noise components are strongly suppressed at sideband frequencies *ω*_0_ ± Ω, Ω ≪ *ω*_*s*_, and all the energy at these frequencies goes into the arm cavity mode $\widehat a$. For large values of Ω, the noise becomes resonant inside the SE cavity as well, reaching its resonance maximum at *ω*_*s*_, as shown in Fig. 2b. It is this particular resonant structure of the coupled system that we take advantage of to enhance the sensitivity of the detector at high frequencies as follows.

We propose to place an optical parametric amplifier, e.g., a *χ*^(2)^ nonlinear crystal, inside the SE cavity. The parametric process amplifies the fluctuations in one quadrature of the mode $\widehat a_q$ and suppress the fluctuations in its conjugate counterpart. Depending on the sideband frequency Ω, the amplification strength varies due to the presence of the coupled cavity structure. At frequencies around *ω*_0_, the excitation of mode $\widehat a_q$ is suppressed, so the parametric process is inefficient, and almost no squeezing is produced. At the same time, the SE cavity is resonant for higher frequencies Ω ~ *ω*_*s*_, so the crystal produces a high squeeze factor. The suppression of shot noise at frequencies 0 ≪ Ω ≪ *ω*_*s*_ happens exactly at the same rate as the reduction in the signal amplification due to the detector bandwidth (see Fig. 2c). The two processes compensate each other, and the SNR remains constant; thus, the bandwidth is expanded (see Fig. 2d).

The quantum expansion effect can be demonstrated in more detail by formulating a complete Hamiltonian of the model two-mode system (for a general analysis of the system, see the Supplementary Material):

1 $$\widehat H = \widehat H_0 + \widehat H_{{\mathrm{int}}} + \widehat H_\gamma + \widehat H_x - F_{{\mathrm{GW}}}x$$

2 $$\widehat H_0 = \hbar \omega _0\widehat a^\dagger \widehat a + \hbar \omega _0\widehat a_q^\dagger \widehat a_q$$

3 $$\widehat H_{{\mathrm{int}}} = \hbar \omega _s\widehat a_q^\dagger \widehat a + \frac{1}{2}\hbar \kappa \beta e^{-2i\omega _0t}\widehat a_q^\dagger \widehat a_q^\dagger e^{i\phi } + h.c.$$

4 $$\widehat H_\gamma\,=\,i\hbar \sqrt {2\gamma } \mathop {\int }\limits_{ - \infty }^\infty \left( {\widehat a_q^\dagger (\omega )\widehat a_{{\mathrm{in}}}(\omega ) - \widehat a_{{\mathrm{in}}}^\dagger (\omega )\widehat a_q(\omega )} \right)d\omega$$

5 $$\widehat H_x\,=\,- \widehat F_{rp}\widehat x\,=\,- \hbar G_0\widehat a^\dagger \widehat a\widehat x$$

where $\widehat a,\widehat a_q$ are the arm cavity and SE cavity modes, respectively, and *ω*_0_ is their natural resonance frequency; $\omega _s\,=\,c\sqrt {T_{{\mathrm{ITM}}}/(4L_{{\mathrm{SE}}}L_{{\mathrm{arm}}})}$ is the coupling rate between two cavities, *T*_ITM_ is the transmission of the front mirror of the arm cavity; *L*_SE_, *L*_arm_ are the lengths of the SE and arm cavity, respectively; *γ* = *cT*_SE_/(4*L*_SE_) is the coupling rate of the SE mode to the continuum of input modes $\widehat a_{{\mathrm{in}}}$; *x* is the displacement of the test mass partially in reaction to the GW tidal force *F*_GW_; the mirror motion *x* is coupled via the radiation-pressure force $\widehat F_{{\mathrm{rp}}}$ to the cavity mode with strength *G*_0_ = *ω*_0_/*L*_arm_; and *κ* is the coupling strength due to crystal nonlinearity under a second harmonic pump field with amplitude *β* and phase *ϕ*. The pump field is assumed to be classical, and its depletion is neglected. Quantum expansion affects only the high-frequency sensitivity, which is dominated by shot noise. This justifies us to ignore in this simple model the effects of the quantum radiation pressure on the dynamics of the test mass, effectively assuming an infinite mass of the mirrors. The displacement of the mirrors *x* in this approximation is caused only by the GW strain *h*_0_ = *x*/*L*_arm_. In addition, note that the expression for the coupling frequency *ω*_*s*_ only applies when *ω*_*s*_ ≪ *ω*_FSR_ ≡ *c*/(2*L*_arm_) and has to be modified when the neighboring longitudinal resonances of the arm cavity are taken into account.

### Input-output relation

The light field in the coupled system can be expressed in terms of the input fields by solving the Hamiltonian above. We write the input-output relations for the amplitude and phase quadrature of the light (denoted by the upper indices (1, 2) correspondingly), representing the field leaving the detector $\widehat a_{{\mathrm{out}}}^{(1)}$, the field inside the SE cavity $\widehat a_q^{(2)}$, and the field inside the arm cavity $\widehat a^{(1)}$ in terms of the input noise fields $\widehat a_{{\mathrm{in}}}^{(1,2)}$:

6 $$\begin{array}{lcc}\widehat a_{{\mathrm{out}}}^{\left( 1 \right)}\left( {\mathrm{\Omega }} \right) = \widehat a_{{\mathrm{in}}}^{\left( 1 \right)}\left( {\mathrm{\Omega }} \right)\frac{{\left( {\gamma - \chi } \right){\mathrm{\Omega }} + i\left( {{\mathrm{\Omega }}^2 - \omega _s^2} \right)}}{{\left( {\gamma + \chi } \right){\mathrm{\Omega }} - i\left( {{\mathrm{\Omega }}^2 - \omega _s^2} \right)}} \\ + \, h_0\left( {\mathrm{\Omega }} \right)\frac{{2iG\sqrt \gamma \omega _s}}{{\left( {\gamma + \chi } \right){\mathrm{\Omega }} - i\left( {{\mathrm{\Omega }}^2 - \omega _s^2} \right)}}\end{array}$$

7 $$\begin{array}{lcc}\widehat a_q^{\left( 2 \right)}\left( {\mathrm{\Omega }} \right) = \widehat a_{{\mathrm{in}}}^{\left( 2 \right)}\left( {\mathrm{\Omega }} \right)\frac{{\sqrt {2\gamma } {\mathrm{\Omega }}}}{{\left( {\gamma + \chi } \right){\mathrm{\Omega }} - i\left( {{\mathrm{\Omega }}^2 - \omega _s^2} \right)}}\\\,\,\,\,\,\,\,\,\,\,\,\,\,\,\,\,\,\,\,\,\,\,\,\,\, + \, h_0\left( {\mathrm{\Omega }} \right)\frac{{iG\omega _s}}{{\left( {\gamma + \chi } \right){\mathrm{\Omega }} - i\left( {{\mathrm{\Omega }}^2 - \omega _s^2} \right)}}\end{array}$$

8 $$\widehat a^{\left( 1 \right)}\left( {\mathrm{\Omega }} \right)\,=\,\widehat a_{{\mathrm{in}}}^{\left( 2 \right)}\left( {\mathrm{\Omega }} \right)\frac{{i\sqrt {2\gamma } \omega _s}}{{\left( {\gamma - \chi } \right){\mathrm{\Omega }}-i\left( {{\mathrm{\Omega }}^2 - \omega _s^2} \right)}}$$

where we linearized the system dynamics and introduced an effective parametric gain *χ* = *κβ* , effective signal coupling strength $G\,=\,\sqrt {2P_cL_{{\mathrm{arm}}}\omega _0/(\hbar c)}$ and optical power inside the arm cavity $P_c = \hbar \omega _0\bar a^2$, with $\overline a$ being an average amplitude of the mode $\widehat a$. The phase of the pump field was chosen such that the parametric process squeezes the signal quadrature inside the SE cavity, i.e., *ϕ* = *π*/2. Several features are provided by these equations. First, when we remove the crystal (i.e., *χ* = 0) in the typical operational range of GWOs Ω ≪ *ω*_*s*_, the input-output relation Eq. (6) reduces to the standard relation for a baseline GWO^43,44^, with the detection bandwidth given by $\gamma _{{\mathrm{baseline}}} = \omega _s^2/\gamma = cT_{{\mathrm{ITM}}}/(T_{{\mathrm{SE}}}L_{{\mathrm{arm}}})$. Second, the noise term in Eq. (7) is strongly suppressed at zero sideband frequency, as we described above in the example with two coupled modes: $\widehat a_q^{(2)}(0) = h(0)G/\omega _s$; therefore, virtually no squeezing is produced at low frequencies. The noise in the output in Eq. (6) at low frequencies is defined by the vacuum field reflected directly off the SE mirror. Third, when the sideband frequency matches the normal mode frequency, Ω = *ω*_*s*_, the signal mode takes the form $\widehat a_{{\mathrm{out}}}^{(1)}(\omega _s) = \widehat a_{{\mathrm{in}}}^{(1)}(\gamma - \chi )/(\gamma + \chi ) + 2ih_0(\omega _s)G\sqrt \gamma /(\gamma + \chi )$. This equation shows that for a parametric gain close to the threshold (*χ* → *γ*), the noise term becomes almost infinitely squeezed^19^, but the signal becomes deamplified by at most a factor of 2. Despite the signal deamplification, ideally, the SNR in this case can become infinite, as we show below by computing the sensitivity of the quantum-expanded observatory.

### Sensitivity spectrum

The noise spectral density of the GWO with a quantum expander, normalized to a GW strain amplitude *h* of unity, can be obtained from Eqs. (6–8) and further approximated in the typical regime where GWOs operate, *γ* ≫ *ω*_*s*_ ≫ Ω, as follows:

9 $$\begin{array}{lcc}S_h\left( {\mathrm{\Omega }} \right) = \frac{{\hbar c}}{{8\omega _0L_{{\mathrm{arm}}}P_c}}\frac{{\left( {{\mathrm{\Omega }}^2 - \omega _s^2} \right)^2 + \left( {\gamma - \chi } \right)^2{\mathrm{\Omega }}^2}}{{\gamma \omega _s^2}}\\\,\,\,\,\,\,\,\,\,\,\,\,\,\,\,\,\,\,\,\, \approx \frac{{\hbar c}}{{8\omega _0L_{{\mathrm{arm}}}P_c}}\frac{{\gamma _q^2 + {\mathrm{\Omega }}^2}}{{\gamma \omega _s^2}}\left( {\gamma - \chi } \right)^2\end{array}$$

with the new detection bandwidth defined as $\gamma _q = \omega _s^2/(\gamma - \chi )$. Without the quantum expansion, *χ* = 0, the baseline sensitivity decreases with increasing frequency, limited by the detector’s bandwidth $\gamma _{{\mathrm{baseline}}} = \omega _s^2/\gamma$:

10 $$\begin{array}{lcc}S_h^{{\mathrm{baseline}}}\left( {\mathrm{\Omega }} \right) = \frac{{\hbar c}}{{8\omega _0L_{{\mathrm{arm}}}P_c}}\frac{{\left( {{\mathrm{\Omega }}^2 - \omega _s^2} \right)^2 + \gamma ^2{\mathrm{\Omega }}^2}}{{\gamma \omega _s^2}}\\\,\,\,\,\,\,\,\,\,\,\,\,\,\,\,\,\,\,\,\,\,\,\,\,\,\,\,\,\,\,\,\,\,\, \approx \frac{{\hbar c}}{{8\omega _0L_{{\mathrm{arm}}}P_c}}\frac{{\gamma _{{\mathrm{baseline}}}^2 + {\mathrm{\Omega }}^2}}{{\gamma \omega _s^2}}\gamma ^2\end{array}$$

The detection bandwidth *γ*_*q*_ can ideally be expanded infinitely (in the two-mode approximation) by a factor of *γ*/(*γ* − *χ*) → ∞ when the squeezing approaches the threshold point *χ* = *γ*. At this point, the sensitivity is given by

11 $$S_h\left( {\mathrm{\Omega }} \right)\,=\,\frac{{\hbar c}}{{8\omega _0L_{{\mathrm{arm}}}P_c}}\frac{{\omega _s^2}}{\gamma }$$

which is approximately frequency independent under Ω ≪ *ω*_*s*_ as a result of the expanded bandwidth *γ*_*q*_. In reality, even in the lossless case, the bandwidth is still limited by the next longitudinal resonance of the arm cavity and the detector’s reduced response when the detector’s arm length is comparable to the gravitational wavelength.

**Fig. 3:**
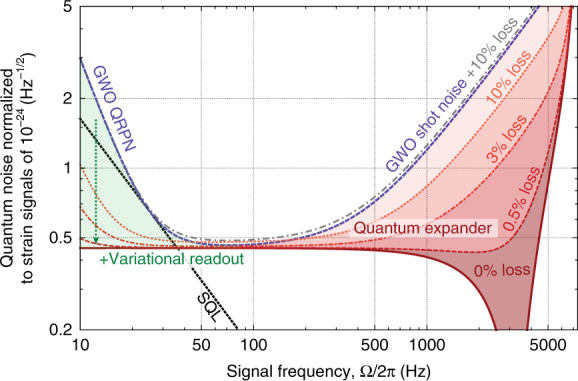
Effect of the quantum expander on the detector’s sensitivity to gravitational-wave strain *S*_*h*_(*f*), in combination with the variational readout^17^. The bandwidth of the semiclassical gravitational wave observatory (GWO, blue dashed line) is expanded by the squeezing operation inside the detector at high frequencies (solid red line, red shading). The effect deteriorates once quantum decoherence due to optical loss is introduced (different shades of red for the quantum expander, gray dot-dashed line for the semiclassical GWO). At low frequencies, quantum noise remains unaffected by quantum expansion, which enables the use of the variational readout (green shading) for evading the quantum radiation-pressure noise (QRPN). The efficiency of the variational readout is also affected by the optical loss, which leads to the loss of correlations between the two quadratures of the light field, resulting in a reduction in the sensitivity at low frequencies, as shown by dashed red lines. The boundary where the QRPN becomes equal to the shot noise at different light powers, known as the standard quantum limit (SQL), is plotted in black dots. The parameters used for plotting are based on the benchmark parameter set for the 3rd generation of GWOs: optical wavelength *λ* = 1550 nm; light power inside the arm cavity *P*_*c*_ = 4 MW; arm cavity length *L*_arm_ = 20 km; SE cavity length *L*_SE_ = 56 m; mirror mass *m* = 200 kg; input mirror power transmission *T*_ITM_ = 0.07; and SE mirror power transmission *T*_SE_ = 0.35.

The effect of the quantum expander on a baseline GWO is shown in Fig. 3. To produce this figure, we compute the sensitivity based on the transfer matrix approach (as presented in the Supplementary Material), which better describes the high-frequency behavior in the longer detectors, i.e., when *ω*_*s*_ ~ *ω*_FSR_. It also takes into account the effects of quantum radiation pressure noise, quantum decoherence (see the Discussion section for more details), the next free spectral ranges of the cavities and the response function of the detector to gravitational waves.

### Quantum Cramer-Rao bound

The sensitivity of any GWO is ultimately limited by its QCRB $S_{h}^{\mathrm{QCRB}}(\Omega)$^15^. The conditions for reaching this bound are that (i) the quantum radiation pressure noise is evaded, and (ii) the upper and lower optical sidebands generated by the GW are equal in amplitude^15^. Naturally, there is also a typical requirement of absence of optical decoherence and technical noises. The quantum expander configuration does not affect the QRPN and allows satisfying condition (i) at low frequencies by well-known back-action evading techniques (e.g., variational readout, see the Discussion below). We prove that condition (ii) is satisfied by directly computing the QCRB in the case of GW detectors, defined as follows:^15^

12 $$S_h^{\rm{QCRB}}(\Omega ) = \frac{{{\hbar ^2}}}{{2L_{\rm{arm}}^2{S_{\rm{FF}}}(\Omega )}} = \frac{{\hbar c}}{{4{\omega _0}{L_{\rm{arm}}}{P_c}}}\frac{1}{{{S_{\rm{aa}}}(\Omega )}}$$

where *S*_FF_(Ω) is the single-sided spectrum of the radiation-pressure force $\widehat F_{{\mathrm{rp}}}$ and *S*_aa_(Ω) is the noise spectrum of the arm cavity field, which one can compute from Eq. (9):

13 $$S_{aa}\left( {\mathrm{\Omega }} \right) = \frac{{2\gamma \omega _s^2}}{{\left( {\gamma - \chi } \right)^2{\mathrm{\Omega }}^2 + \left( {{\mathrm{\Omega }}^2 - \omega _s^2} \right)^2}}$$

Therefore, the limit on the sensitivity is given by the QCRB in the following form:

14 $$S_x^{{\mathrm{QCRB}}}\left( {\mathrm{\Omega }} \right) = \frac{{\hbar c}}{{4\omega _0L_{{\mathrm{arm}}}P_c}}\frac{{\left( {{\mathrm{\Omega }}^2 - \omega _s^2} \right)^2 + \left( {\gamma - \chi } \right)^2{\mathrm{\Omega }}^2}}{{2\gamma \omega _s^2}}$$

which is identical to Eq. (9). The sensitivity becomes unbounded (QCRB goes to zero) at the parametric threshold *χ* = *γ* at frequency Ω = *ω*_*s*_.

This calculation demonstrates that the quantum expander strongly reduces the QCRB at high frequencies compared to the baseline GWO and that the expanded detector does reach its lowered QCRB (in the case of the typical assumption of zero photon loss).

## Discussion

### Quantum decoherence

Non-classical light is sensitive to decoherence, i.e., to optical loss, which destroys the inherent quantum correlations^45^. Losses occur inside the detector, as well as on the readout and have multiple contributions. Any squeezed light application and QRPN suppression technique is limited by optical loss, and the proposed scheme is not an exception. The quantum expander relies on squeezing operation inside the interferometer to compensate for the decrease in the signal amplification due to the finite cavity linewidth. The higher the squeeze factor is, the more it is susceptible to optical loss. The effect of different readout losses is shown in Fig. 3. In the current generation of GWOs, the optical readout loss is on the order of 10%^46^, and in next observatory generation, 3–5% might be achievable^47^. With advanced techniques, which have been proposed^48,49^ but have yet to be explored experimentally, the readout loss could conceivably be reduced to as small as 0.5%. Introducing a nonlinear crystal inside the detector will increase the internal loss; however, there is no fundamental reason why such a crystal should have a bulk or surface absorption higher than that of the interferometer central beam splitter. Fortunately, the crystal needs to be placed at a position where the light power is comparatively low. There are other technical issues associated with placing a crystal inside the detector, such as the need for intense second-harmonic pump light, the requirements on the crystal size being larger than the beam diameter and the potential influence of the parametric amplification process on the interferometer’s control and stabilization systems. These issues are crucial for the practical detector design, yet rather technical in nature, so their detailed discussion can be found in the Supplementary Material. We believe that the added loss due to a crystal can be relatively small (see, e.g., the discussion in ref. ^39^) and the additional technical challenges can be overcome, and we consider our work to be a strong motivation for detailed experimental research and development.

### Combination with external-squeezing injection

All observatories of the current generation are already operating with external-squeezing injection. When the quantum expander is combined with external-squeezing injection, the overall squeeze factor at high frequencies increases further. This makes the requirements for low optical loss more stringent. The benefit from quantum expansion in combination with external squeezing depends not only on the amount of loss but also on the places where it occurs. There exists an optimal parametric gain in the quantum expander that maximizes the sensitivity by balancing the signal deamplification in the parametric process and squeeze factor^39^. Ultimately, every specific design of the detector has to be optimized with respect to optical parameters to be able to maximally benefit from quantum expansion. An in-depth discussion of the physical and technical aspects of combining the quantum expander with external-squeezing injection can be found in the Supplementary Material.

We envision the quantum expander to benefit future generations of GWOs when technological progress allows lowering of optical losses and detectors become longer and overall more sensitive, e.g., in the extensions of the third generation of observatories (Einstein Telescope and Cosmic Explorer) and beyond.

### Combination with other QND techniques

Quantum-expanded signal extraction will further reduce the shot noise at high frequencies without affecting the established improvement factor from external squeezing. The quantum noise at *low* frequencies (QRPN) will remain unchanged. This distinguishes our approach from other designs targeting the high-frequency sensitivity^44,50,51^. The QRPN can be suppressed independently using already developed approaches using frequency-dependent squeezing, variational readout or quantum non-demolition measurements^17,36–38^. In Fig. 3, we illustrate this point by combining the quantum expander with the variational readout technique, which requires an additional filter cavity (assumed lossless here) on the output of the GWO.

### Potential astrophysical applications

**Fig. 4:**
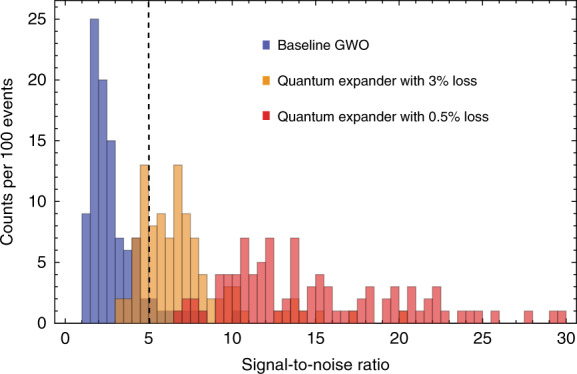
Histogram for the SNR of the loudest event for 100 realizations in the Monte-Carlo simulation. Blue bins represent the SNR of our baseline GWO. Orange and red bins are associated with quantum expanders with a total loss of ~3 and 0.5%, respectively. The black dashed line indicates a detection threshold (SNR = 5). We used the equation of state in refs. ^52,53^, and the binary merger rate is taken to be *R* = 1.54 Mpc^−3^Myr^−1^. The mass distribution for each neutron star in the binary is taken to be Gaussian and centered around 1.33 $M_ \odot$.

Currently, all GWOs maximize the SNR at frequencies around 100 Hz, where signals of compact binary inspirals can be observed. Merger and post-merger signals from binary neutron stars are expected to be at frequencies of ~1–3 kHz, where the sensitivity of the detectors significantly deteriorates due to the detection bandwidth. Quantum expansion of the detection bandwidth allows an increase in the sensitivity exactly at these frequencies, as shown in Fig. 3. To illustrate the potential of the quantum expander, we compute the SNR of a particular model of the post-merger signal^52,53^ (see the details in the Supplementary Materials) and demonstrate the improvement in detection rates on the histogram in Fig. 4: from a 9% chance of a single loud event surpassing the detection threshold after a full year of data acquisition in a baseline GWO, to ~76% and almost 100% for quantum-expanded detectors with 3 and 0.5% optical loss, respectively.

We envision the quantum expander as a potential tool allowing GW observatories to contribute to a better understanding of the physics of ultra-dense quantum matter in neutron stars and astrophysics of compact objects in general. We anticipate that other metrological^54,55^, as well as optomechanical^56,57^ experiments will benefit from our approach of using a coupled-cavity system with an internal parametric amplifier for bandwidth expansion.

## Materials and methods

The Hamiltonian of the quantum expander, presented in the Results section, is constructed based on the input-output relations for the noise and the signal^58,59^. For this derivation, we assume the effects of radiation-pressure noise and optical losses to be negligible, although the full model used to plot Fig. 3 includes both of these effects and is presented in the Supplementary Materials. The results of the derivation of the input-output relations, which we present in the full extent in the Supplementary Materials, allow us to obtain the shot-noise limited output field containing the noise *a*_*n*_(Ω) and signal terms *X*_out_(Ω):

15 $$a_{{\mathrm{out}}}^{(c)}\left({\mathrm{\Omega }}\right) = a_n\left( {\mathrm{\Omega }} \right) + X_{{\mathrm{out}}}\left( {\mathrm{\Omega }} \right)$$

16 $$\begin{array}{lcc}a_n\left({\mathrm{\Omega}}\right) = - \frac{{e^{2i\varphi }e^{2i\Omega \tau _{{\mathrm{SE}}}}(e^{2i\Omega \tau _{{\mathrm{arm}}}} - R_i) + e^{2q}(e^{2i\Omega \tau _{{\mathrm{arm}}}}R_i - 1)}}{{e^{2q}(e^{2i\Omega \tau _{{\mathrm{arm}}}}R_i - 1) + e^{2i\varphi }e^{2i\Omega \tau _{{\mathrm{SE}}}}(e^{2i\Omega \tau _{{\mathrm{arm}}}} - R_i)R_s}}\\\,\,\,\,\,\,\,\,\,\,\,\,\,\,\,\,\,\,\,\,\,\,\,\,\,\,\,a_{{\mathrm{in}}}^{(c)}\left( {\mathrm{\Omega}} \right)\end{array}$$

17 $$\begin{array}{lcc}X_{{\mathrm{out}}}\left( {\mathrm{\Omega }} \right)\,=\,\frac{{2ik_p\bar ae^{i\varphi }e^{i\Omega \tau _{SE}}e^{i\Omega \tau _{{\mathrm{arm}}}}e^qT_iT_s}}{{e^{2q}(e^{2i\Omega \tau _{{\mathrm{arm}}}}R_i\,-\,1)\,+\,e^{2i\varphi }e^{2i\Omega \tau _{{\mathrm{SE}}}}(e^{2i\Omega \tau _{{\mathrm{arm}}}} - R_i)R_s}}\\\,\,\,\,\,\,\,\,\,\,\,\,\,\,\,\,\,\,\,\,\,\,\,\,\,\,\,\,x\left( {\mathrm{\Omega}} \right)\end{array}$$

where $R_{i,s}\,=\,\sqrt {R_{{\mathrm{ITM,SE}}}} ,T_{i,s} = \sqrt {T_{{\mathrm{ITM,SE}}}}$ are, respectively, the amplitude reflectivity and transmissivity of the input test mirror and signal-extraction mirror; *q* is an amplification factor on the single pass through the crystal; *τ*_arm,SE_ = *L*_arm,SE_/*c* is the single trip time in arm cavity of length *L*_arm_ and SE cavity of length *L*_SE_, with *c* being the speed of light; *φ* = *π*/2 is the tuning of the SE cavity with respect to the arm cavity; *x* = *h*_0_*L*_arm_ is a small displacement of the end mirror due to the GW strain *h*_0_; $\overline a$ is the average amplitude of field inside the arm cavity and *k*_*p*_ is the wave vector of the carrier light field.

These expressions can be simplified by making several approximations that are realistic for GWOs. We assume that Ω*τ*_arm_ ≪ 1 and Ω*τ*_SE_ ≪ 1, so $e^{i\Omega \tau _{{\mathrm{arm,SE}}}}\,\approx\,1\,+\,i\Omega \tau _{{\mathrm{arm}},{\mathrm{SE}}}$; *T*_*i,s*_ ≪ 1, so $R_i \approx 1 - T_i^2/2\,=\,1\,-\,2\gamma _{{\mathrm{arm}}}\tau _{{\mathrm{arm}}}$ and $R_s\,\approx\,1\,-\,T_s^2/2\,=\,1\,-\,2\gamma \tau _{{\mathrm{arm}}}$, where *γ*_arm_, *γ* are the arm cavity and the signal-extraction cavity linewidth, respectively; and the single-pass optical gain is small: *q* ≪ 1, so *e*^*q*^ ≈ 1 + *q* = 1 + *χτ*_SE_, where *χ* is an effective parametric gain. In these assumptions, we obtain Eq. (6) for the output noise and signal. This allows us to construct the Hamiltonian presented in Eqs. (1–5).

We would like to point out the limits of this approximation: it is valid only until coupling and signal frequencies are much smaller than the free spectral range of the arm cavity: Ω, *ω*_*s*_ ≪ *ω*_FSR _≡ *c*/2*L*_arm_. This condition sets a limit on the transmissivity of the ITM: $T_i^2 \ll L_{{\mathrm{SE}}}/L_{{\mathrm{arm}}}$, and restricts the applicability of the derived simplified equations to a detector with a relatively short arm length (e.g., Advanced LIGO). A longer detector (such as the baseline GWO chosen as a reference in Figs. 2–4) would require a more sophisticated expression with the higher longitudinal resonances of the arm cavity taken into account. The assumption of a small transmission of the SE mirror is often not valid in real designs, leading to additional contributions in the noise spectrum. We perform the full analysis, which avoids these limitations, in Section 5 of the Supplementary Materials.

## Supplementary information

Supplemetary materials

## Data Availability

The code used to produce the figures is available by request to the corresponding author.
