# Supplementary material for: Quantum expander for gravitational-wave observatories
Source: Light Sci Appl. 2019 Dec 11;8:118. doi: 10.1038/s41377-019-0230-2 (PMC6904558; doi:10.1038/s41377-019-0230-2)
Supplement: Supplementary file 1 — Supplemetary materials [file 41377_2019_230_MOESM1_ESM.pdf]

Supplementary Materials for

Quantum expander for gravitational-wave observatories

Mikhail Korobko,[1*] Yiqiu Ma,[2] Yanbei Chen,[2] Roman Schnabel[1]

[1]Institut für Laserphysik und Zentrum für Optische Quantentechnologien, Universität Hamburg
Luruper Chaussee 149, 22761 Hamburg, Germany
[2]Theoretical Astrophysics 350-17, California Institute of Technology, Pasadena, California 91125, USA

*E-mail: mkorobko@physnet.uni-hamburg.de

This PDF file includes:

Supplementary Text

Table 1

Figs. 1 to 4

Contents

S1 Experimental feasibility **2**

S1.1 Optical loss 2

S1.2 Crystal inside the detector 6

S2 Astrophysical analysis **8**

S3 Input-output relations **9**

S4 Hamiltonian approach **13**

S5 Transfer matrix approach to full description **16**

S5.1 Input-output relations 16

S5.2 Radiation pressure 20

S5.3 Detection 21

S5.4 Filter cavities 23

Supplementary Text

In the supplementary text we discuss the experimental feasibility of the quantum expander, with emphasis on the optical loss sources and the beneficial combination of the quantum expander with external squeezing of the light's quantum noise; derive the equations in the main text based on the simplified input-output relations and on the Hamiltonian description of the system; give details on the astrophysical implications of use of quantum expander; provide details of derivation of the full spectral density of the detector with various imperfections based on the optical transfer matrix approach.

S1 Experimental feasibility

In this section we discuss some of the issues of the experimental feasibility. We indicate the main sources of loss and their contribution into the resulting sensitivity, and analyze the achievable benefit from quantum expansion when combined with external squeezed-light injection.

S1.1 Optical loss

As we discuss in the main text, our quantum expander creates squeezing at high frequencies to counteract the effect of the detector's bandwidth. When combined with external squeezing, the

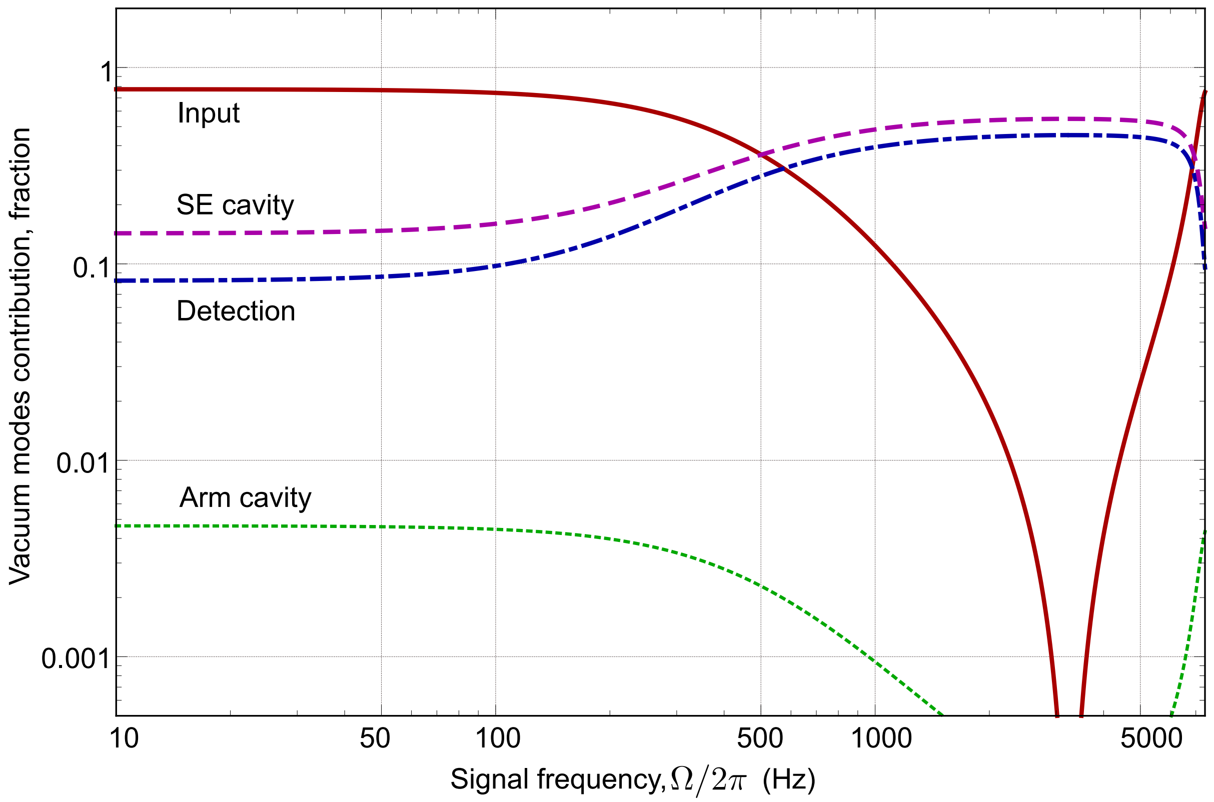

Fig. S1: Relative contribution of different vacuum modes (optical loss) to the overall sensitivity of the detector versus frequency. Input vacuum mode (solid red) defines the main sensitivity level. The rest arises from various sources of loss: loss inside the SE cavity (dashed magenta), detection loss (dot-dashed blue) and arm cavity loss (dotted green). The parameters are taken according to Table S1: internal loss is 1500 ppm single-trip, detection loss is 1%, transmission of the end mirror is 100 ppm (increased relative to Table S1 to emphasize the smallness of its influence on the sensitivity)

quantum expander produces a high amount of squeezing at high frequencies, which imposes strict demands on reducing the optical losses. The losses occur inside the detector: inside the arm cavity, and inside the SE cavity; as well as on the readout train: from the SE mirror to the detector. The external squeezing additionally suffers from injection loss. On Fig. S1 we show the contribution of different sources of loss as a function of frequency. We note that the detection loss and loss inside the SE cavity are the most important contributions. The detection loss currently is rather high, but the way to mitigate this loss by parametric amplification was

proposed by Caves (*1*) and recently re-investigated experimentally (*2*). The idea of this approach is to amplify both the signal and the noise by the same amount before it experiences loss, such that the resulting noise is much above vacuum uncertainty, and the loss does not affect it significantly. The simplest example of it is detecting some signal G embedded in squeezed vacuum, with detection efficiency η:

$$S = \eta(e^{-2r} + G^2)e^{2q} + 1 - \eta, \tag{1}$$

where r is the squeeze factor and q is the Caves' amplification factor. The signal-to-noise ratio is given by $\mathrm{SNR} = \eta e^{2q} \left(1 - \eta(1 - e^{-2r}e^{2q})\right)^{-1}$. Without amplification, $q = 0$, in the limit of large squeezing $e^{-2r} \approx 0$ the SNR is limited to $\mathrm{SNR}_{q=0} \leq \eta(1-\eta)^{-1}$. When the amplification is large, $q \to \infty$, the SNR becomes independent on the loss: $\mathrm{SNR}_{q\to\infty} = e^{2r}$, and only benefits from initial squeezing. The only source of detection loss that cannot be mitigated by Caves' amplification is the loss in the Faraday isolator used for injecting external squeezing. We assume this to be a limitation in the detection loss, which corresponds to the 0.5% (*3*) mentioned in the main text.

Internal loss will be increased due to the additional optical surfaces of the nonlinear crystal and the absorption of the crystal. While the actual contribution to the loss from such a crystal requires a separate investigation, we give an estimate based on the squeezing cavity design for the table-top experiments. If the PPKTP crystal will be used, it's absorption is ~ 100 ppm per cm depending on wavelength (*4*); the surfaces of the crystal will have to be coated with anti-reflecting coating to minimize the scattering loss. We estimate that the current standard technology can bring this added loss on the level of 200–500 ppm in single-pass.

We would like to emphasize, that not every configuration of the GWO will be able to get a significant benefit from quantum expansion when the external squeezing is in use. Depending on the amount of loss, and amount of external squeezing injected, the benefit will vary. The

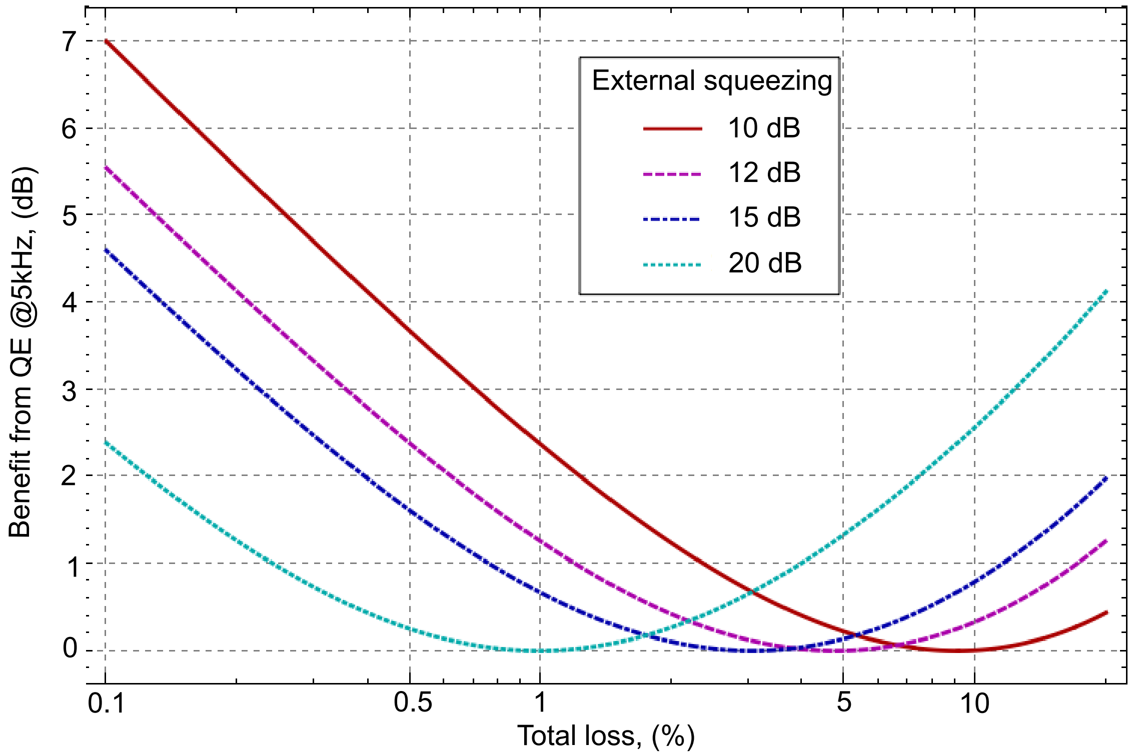

Fig. S2: An improvement in the sensitivity of the detector by quantum expander, relative to the detector with external squeezing injection, depending on the amount of total loss (internal and readout). The higher is the external squeezing, the more stringent is the loss requirement for being able to benefit from using the quantum expander. The sensitivity depends in a non-trivial way on the losses, which is reflected in the benefit from QE shown on the figure.

reason is an additional de-amplification of the signal in the quantum expander. When the loss is high, the squeezing of the noise by quantum expander in addition to external squeezing might be not significant. However, the parametric process inside the detector reduces the signal, hence the signal-to-noise ratio might even become reduced compared to the detector without quantum expander, if the sub-optimal parametric gain is chosen. There always exists an optimal gain, for which the benefit is maximal. If the loss is high, it might be optimal to amplify the signal (and anti-squeeze the noise), similar to the Caves' amplification discussed above. We demonstrate possible improvements to the sensitivity in Fig. S2. We note, that this specific design is based on

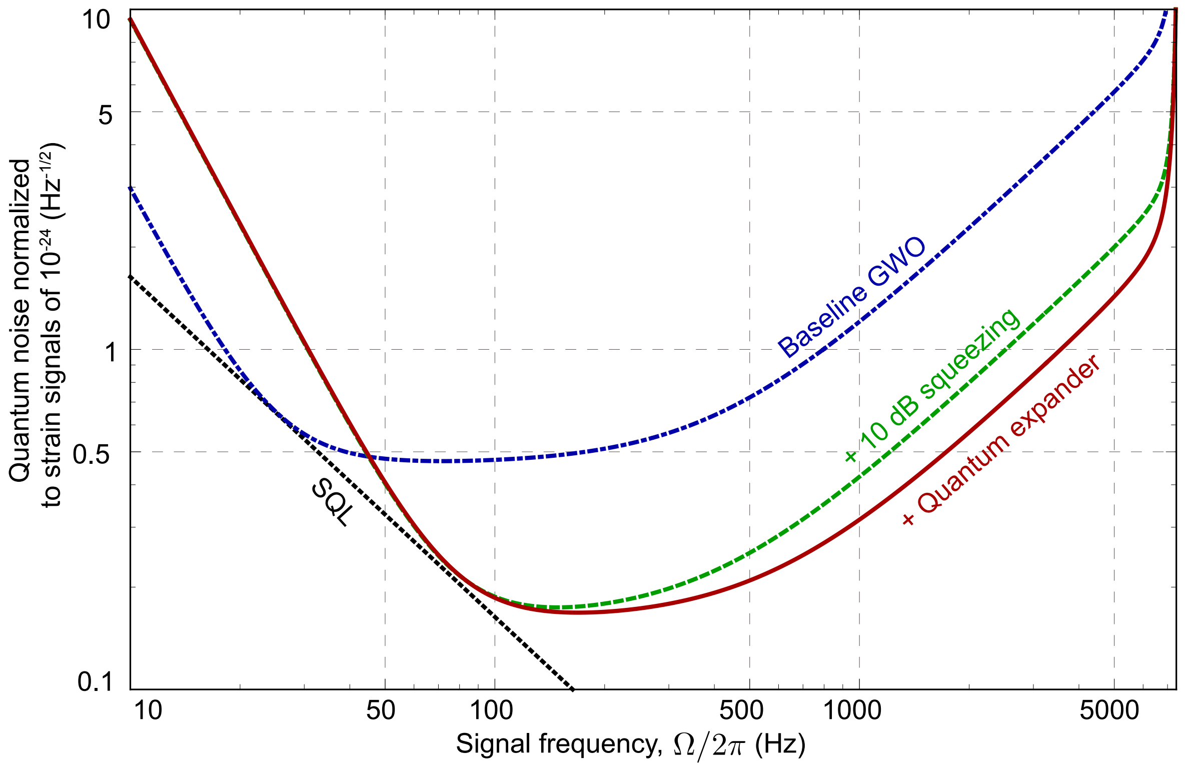

Fig. S3: An example of sensitivity improvement in a particular design of a detector with 1% of total loss and 10 dB external squeezing injection, the parameters are given in Table S1.

the benchmark parameters adopted by the LIGO-Virgo Collaboration, as presented in Table 1, and corresponds to the sensitivity as given in Fig. S3. In reality, the benefit from quantum expansion can be increased by optimizing the optical design (e.g. SE cavity length and mirrors' reflectivities). The optimized sensitivity given by the quantum expander is a topic of future studies.

S1.2 Crystal inside the detector

There are several issues to be taken into account with placing the crystal inside the SE cavity.

First, the aperture of the crystal has to be large to avoid beam clipping at the edges of the crystal. Currently the diameter of the beam inside the SE cavity is $\sim 2\,\mathrm{cm}$ (*6*), with the focal

parameter	description	Baseline GWO	AdvLIGO
λ	optical wavelength	1550 nm	1064 nm
$P_{\rm arm} = P_c/2$	arm cavity light power	4 MW	840 kW
$L_{\rm arm}$	arm cavity length	20 km	4 km
m	mirror mass	200 kg	40 kg
$L_{\rm SE}$	SE cavity length	56 m	56 m
$T_{\rm ITM}$	input mirror power transmission	0.07	0.014
$T_{\rm SE}$	SE mirror power transmission	0.35	0.35
$T_{\rm ETM}$	end mirror power transmission	5 ppm	5 ppm
e^{2r}	external squeezing	10 dB	—
λ_s	loss inside SE cavity	1500 ppm	1000 ppm
η	detection efficiency	99%	∼85%

Table S 1: In order to plot the spectral densities in the paper we use the following set of parameters of some baseline GW observatory, without choosing a specific design from many possibilities of a 3-G topologies. We note that our double-cavity model uses effective parameters. In order to use this model for the Michelson topology, an effective light power inside the arm cavity has to be used: $P_c = 2P_{\rm arm}$, where $P_{\rm arm}$ is the power inside the arms of the Michelson topology (*5*)

point outside the SE cavity. For comparison, the aperture of typical PPKTP crystals used in the squeezed-light generation is 1×2 mm (*4*). Either large enough crystals need to be realized or the beam need to be focused inside the SE crystal, which is in principle possible without increasing the number of optics inside the SE cavity.

Second, while absorption and scattering are generally important issues to avoid possible heating and parasitic interferences (*7*), the crystal will not be illuminated by bright light, because the detector is operated with a destructive interference of carrier light in the SE cavity (dark port condition).

Third, the crystal has to be pumped with frequency doubled light of a sufficient intensity, which would require an additional cavity for the pump light, as it is realized in modern table-top squeezing experiments (*8*).

It would also be necessary to deliver the pump to the crystal inside the detector and ensure

good wavefront matching of the pump and the main beam. Both aspects can be achieved in several ways. As the wavelength of the pump is so different from the fundamental wavelength, it is possible to realize optical coatings, such that an additional cavity is formed by the SEM and ITM for the pump (*9*). Alternatively, the pump can be brought in by replacing the existing steering mirrors in the SE cavity with dichroic steering mirrors, being transmissive for the frequency doubled pump. In any case, no additional optics inside the main interferometer would be required.

Fourth, there is a need for additional control of the pump amplitude and phase to ensure the optimal squeeze factor and squeeze angle. Finally, the non-linear crystal might affect the control of the main interferometer, as some of the auxiliary beams could be affected by the parametric process, which has to be taken into account when designing the detector's control scheme.

In conclusion, while a non-linear crystal inside the interferometer is technologically challenging, we do not foresee fundamental problems, and expect our proposal for quantum expansion to motivate the future research and development work in this direction.

S2 Astrophysical analysis

In this section, we give an illustrative example to estimate the capability of using the quantum expanders to detect the gravitational waves radiated by neutron star poster-merger remnants. The method we used here follows the estimation procedure as described in (*10, 11*). We perform a Monte Carlo simulation based on the following assumptions: first, the mass of each individual neutron star in a binary system follows an independent Gaussian distribution centered at $1.33M_\odot$ with variance $0.09M_\odot$. The distributions of angular sky position, inclination and polarisation angles, and the initial phase of the source are assumed to be flat. The searching range is assumed to be $1\,$Gpc and the event rate is taken to be $\approx 1\,\mathrm{Mpc}^{-3}\mathrm{Myr}^{-1}$. Second, the

post-merger waveform is assumed to be a parametrized damped oscillation, which depends on the equation of state of a neutron star, and in frequency domain it is given by the equation:

$$h(f) = \frac{50\text{Mpc}}{\pi d} h_p \frac{Q(2f_p Q\cos\phi_0 - (f_p - 2ifQ)\sin\phi_0)}{f_p^2 - 4iff_pQ - 4Q^2(f^2 - f_p^2)}, \tag{2}$$

where d is the source distance, h_p is the peak value of the wave amplitude, Q is the quality factor of the post-merger oscillation,ϕ_0, f_p are the initial phase and the peak frequency of the waveform, respectively. Among them, h_p, Q, f_p are parametrized by fitting with the results generated by numerical simulation (*12*) and they depend on the choice of equation of states. In the illustrative examples here, we make use of a relatively stiffer equation of state proposed in (*13*), where $Q = 23.3, h_p \approx 5 \times 10^{-22}$, and the peak frequency is given by:

$$f_p = 1\text{kHz}\left(\frac{m_1 + m_2}{M_\odot}\right)\left[a_2\left(\frac{R}{1\text{km}}\right)^2 + a_1\frac{R}{1\text{km}} + a_0\right], \tag{3}$$

where $R = 14.42\,\text{km}$ is the radius of each neutron star, and $m_{1,2}$ are their masses. The parameters a_2, a_1, a_0 take the value of $5.503, -0.5495, 0.0157$, respectively (*13*). We define the signal to noise ratio as:

$$\text{SNR} = \int_{f_{\min}}^{f_{\max}} df \frac{|h(f)|^2}{S_{hh}(f)}, \tag{4}$$

where we take the integration range to be $f_{\min} = 1000\,\text{Hz}$, $f_{\max} = 4000\,\text{Hz}$. We run 100 Monte-Carlo realizations each with 1000 samples, corresponds to one-year observation. We exclude the binaries with total mass larger than $3.45M_\odot$ since they will collapse into a black hole in a very short period of time, less than one period of post-merger oscillation. For each different interferometer parameter set, we selected out the loudest event in each Monte-Carlo realization, set $\text{SNR} = 5$ as a threshold signal-to-noise ratio and produce the Fig. 4 in the main text.

S3 Input-output relations

In this section we derive the sensitivity based on the input-output formalism. For simplicity in this section we ignore the effects of quantum radiation pressure noise and optical losses.

These will be included in the full transfer matrix description in Sec. S5. Based on the obtained equations we give motivation for writing the Hamiltonian of the system in Sec. S4.

Using the perturbation theory, we decompose the light field into a steady-state amplitude with amplitude A_0 and laser carrier frequency ω_0 and a slowly varying noise amplitude $a(t)$ (see details in (*14*)):

$$A(t) = \sqrt{\frac{2\pi\hbar\omega_0}{\mathcal{A}c}}\left[A_0 e^{-i\omega_0 t} + a(t)e^{-i\omega_0 t}\right] + \text{h.c.} \tag{5}$$

$$\hat{a}(t) = \int_{-\infty}^{\infty} \hat{a}(\omega_0 + \Omega)e^{-i\Omega t}\frac{d\Omega}{2\pi}, \tag{6}$$

where $\mathcal{A}$ is the laser beam cross-section area, $\hbar$ is the reduced Plank constant. It is helpful to consider the input-output relations of our system in the 'two-photon formalism' (*15*, *16*), where the amplitude and phase quadrature amplitudes $\hat{a}^{(c)}$ and $\hat{a}^{(s)}$ of the modulation field at frequency Ω are linked to the optical fields $\hat{a}(\omega \pm \Omega)$ via

$$\hat{a}^{(c)}(\Omega) = \frac{\hat{a}(\omega + \Omega) + \hat{a}^{\dagger}(\omega - \Omega))}{\sqrt{2}}, \tag{7}$$

$$\hat{a}^{(s)}(\Omega) = \frac{\hat{a}(\omega + \Omega) - \hat{a}^{\dagger}(\omega - \Omega)}{i\sqrt{2}}. \tag{8}$$

These operators obey the commutation relation

$$[a_x(\Omega), a_x(\Omega')] = [a_y(\Omega), a_y(\Omega')] = 0\,, \tag{9}$$

$$[a_x(\Omega), a_y(\Omega')] = [a_x(\Omega), a_y(\Omega')] = 2\pi i\delta(\Omega + \Omega')\,. \tag{10}$$

We make several simplifications to the notation: as we are primarily interested in the phase quadrature, we will omit index (s) in equations below; we also omit the hats on the operators for brevity, although all the fields are quantised; we consider only the noise fields in the frequency domain, so we don't write that in the equations explicitly: *e.g.* $\hat{a}^{(\mathrm{s})}(\Omega) \to a$.

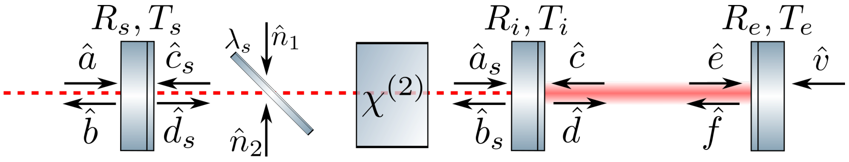

Fig. S4: Quantum fields in the model of a two-cavity system. $R_{s,i,e}, T_{s,i,e}$ are the amplitude reflectivities and transmissivities of the signal extraction, input and end test mirrors correspondingly; a beam-splitter with power reflectivity λ_s represents a source of intra-cavity loss, which causes vacuum noises $\hat{n}_{1,2}$ to enter the system.

The signal we consider is a phase modulation on the light field induced by motion of the mirror with infinite mass caused by an external force. This modulation adds a phase shift on the light reflected off the movable mirror: $E_{\text{refl}} = E_{\text{in}} e^{2ikx(\Omega)} \approx E_{\text{in}}(1 + 2ik_p x(\Omega))$, where k_p is the light's wave vector, $E_{\text{refl,in}}$ are the amplitudes of the reflected and incident light fields, and $x(\Omega)$ is a small mirror displacement. The signal appears only in the equations for the phase quadrature of the light field.

We model the parametric amplification process as a simple linear amplification of amplitude quadrature of the light by some factor e^q, without considering the effects of the parametric pump and the finite size of a crystal. In the full model in Sec. 5 we also will introduce the possibility to tune the amplification quadrature. With this in mind we start with writing down the steady-state input-output relations (*15, 16*) for the quantum fluctuations of the phase quadrature of the light field, for the cavity cavity model depicted of Fig. S4. For the detailed explanation of the approach we refer the reader to the review by Danilishin and Khalili (*14*). We choose the arm cavity to be tuned on resonance, so that for $\Omega = 0$ it has the maximal light power inside.

$$\begin{aligned}
d_s &= T_s a + R_s c_s, && (11)\\
a_s &= d_s e^{-q} e^{i\varphi} e^{i\Omega\tau_{\rm SE}}\,, && (12)\\
b_s &= T_i c + R_i a_s, && (13)\\
c &= d e^{2i\Omega\tau_{\rm arm}} + 2ik_p E x e^{i\Omega\tau_{\rm arm}}\,, && (14)\\
b_s &= -R_i a_s + T_i c\,, && (15)\\
c_s &= b_s e^{-q} e^{i\varphi} e^{i\Omega\tau_{\rm SE}}\,, && (16)\\
b &= -R_s a + T_s c_s\,, && (17)
\end{aligned}$$

where $R_{i,s} = \sqrt{R_{\rm ITM,SE}}, T_{i,s} = \sqrt{T_{\rm ITM,SE}}$ are the amplitude reflectivity and transmissivity of input test mirror and signal-extraction mirror; q is an amplification factor on the single pass through the crystal; $\tau_{\rm arm,SE} = L_{\rm arm,SE}/c$ is the single trip time in arm cavity of length $L_{\rm arm}$ and signal extraction cavity of length $L_{\rm SE}$, with c being the speed of light; $\varphi = \pi/2$ is the tuning of the SE cavity with respect to the arm cavity; x is a small displacement of the end mirror due to the GW signal, E is the large classical amplitude of field inside the arm cavity and k_p is the wave vector of the carrier light field.

We find a solution to these equation, splitting the output b into the noise part b_n and signal $G_{\rm out}$: $b = b_n + X_{\rm out}$.

$$b_n = \mathcal{R}_a(\Omega)a(\Omega) = -\frac{e^{2i\varphi}e^{2i\Omega\tau_{\rm SE}}\big(e^{2i\Omega\tau_{\rm arm}} - R_i\big) + e^{2q}\big(e^{2i\Omega\tau_{\rm arm}}R_i - 1\big)}{e^{2q}\big(e^{2i\Omega\tau_{\rm arm}}R_i - 1\big) + e^{2i\varphi}e^{2i\Omega\tau_{\rm SE}}\big(e^{2i\Omega\tau_{\rm arm}} - R_i\big)R_s}a(\Omega)\,, \quad (18)$$

$$X_{\rm out} = \mathcal{T}(\Omega)x(\Omega) = \frac{2ik_pEe^{i\varphi}e^{i\Omega\tau_{\rm SE}}e^{i\Omega\tau_{\rm arm}}e^{q}T_iT_s}{e^{2q}\big(e^{2i\Omega\tau_{\rm arm}}R_i - 1\big) + e^{2i\varphi}e^{2i\Omega\tau_{\rm SE}}\big(e^{2i\Omega\tau_{\rm arm}} - R_i\big)R_s}x(\Omega)\,, \quad (19)$$

where $\mathcal{R}_a(\Omega), \mathcal{T}(\Omega)$ are the noise and signal optical transfer functions correspondingly.

We can obtain an intuitive expression for these functions by doing several approximations. We assume $\Omega\tau_{\rm arm} \ll 1$, $\Omega\tau_{\rm SE} \ll 1$, so $e^{i\Omega\tau_{\rm arm,SE}} \approx 1 + i\Omega\tau_{\rm arm,SE}$; and $T_{i,s} \ll 1$, so $R_i \approx$

$1 - T_i^2/2 = 1 - 2\gamma_{\rm arm}\tau_{\rm arm}$, $R_s \approx 1 - T_s^2/2 = 1 - 2\gamma\tau_{\rm arm}$, where $\gamma_{\rm arm}, \gamma$ are the arm cavity and the signal-extraction cavity linewidth, respectively; a single-pass optical gain is small: $q \ll 1$, so $e^q \approx 1 + q = 1 + \chi\tau_{\rm SE}$, where χ is an effective parametric gain.

With these approximations equations (18-19) can be simplified to

$$\mathcal{R}_a(\Omega) = \frac{(\gamma - \chi)\Omega + i(\Omega^2 - \omega_s^2)}{(\gamma + \chi)\Omega - i(\Omega^2 - \omega_s^2)}\,, \tag{20}$$

$$\mathcal{T}(\Omega) = -\frac{4ik_pE}{\sqrt{\tau_{\rm arm}}}\frac{\sqrt{\gamma}\omega_s}{(\gamma + \chi)\Omega - i(\Omega^2 - \omega_s^2)}\,, \tag{21}$$

where we defined a sloshing frequency $\omega_s = c\sqrt{T_i^2/(4L_{\rm SE}L_{\rm arm})}$. Notice that these equations correspond to Eq. (6) in the main text. This helps us to construct a Hamiltonian in the next section, which would correspond to this model system.

S4 Hamiltonian approach

In this section we derive the sensitivity of the detector (Eq. (6) of the main text) from the Hamiltonian of the system. The Hamiltonian is based on the input-output formalism, derived in the previous section, where a set of approximations was made. These approximations restrict the analysis to the case when only two modes are taken into account: one in the arm cavity and one in the signal extraction cavity.

$$\hat{H} = \hat{H}_0 + \hat{H}_{\rm int} + \hat{H}_\gamma + \hat{H}_x - F_{\rm GW}x\,, \tag{22}$$

$$\hat{H}_0 = \hbar\omega_0\hat{a}^\dagger\hat{a} + \hbar\omega_0\hat{a}_q^\dagger\hat{a}_q\,, \tag{23}$$

$$\hat{H}_{\rm int} = \hbar\omega_s\hat{a}_q^\dagger\hat{a} + \frac{1}{2}\hbar\kappa\beta e^{-2i\omega_0 t}\hat{a}_q^\dagger\hat{a}_q^\dagger e^{i\phi} + h.c.\,, \tag{24}$$

$$\hat{H}_\gamma = i\hbar\sqrt{2\gamma}\int_{-\infty}^{\infty}\left(\hat{a}_q^\dagger(\omega)\hat{a}_{\rm in}(\omega) - \hat{a}_{\rm in}^\dagger(\omega)\hat{a}_q(\omega)\right)d\omega\,, \tag{25}$$

$$\hat{H}_x = -\hat{F}_{\rm rp}\hat{x} = -\hbar G_0\hat{a}^\dagger\hat{a}\hat{x}\,, \tag{26}$$

where $\hat{a}$, $\hat{a}_q$ are the arm cavity and SE cavity modes, and ω_0 is their natural resonance frequency; $\omega_s = c\sqrt{T_{\rm ITM}/(4L_{\rm SE}L_{\rm arm})}$ is the coupling rate between two cavities, $T_{\rm ITM}$ is the transmission of the front mirror of the arm cavity, $L_{\rm SE}, L_{\rm arm}$ are the lengths of the signal extraction and arm cavity, respectively; $\gamma = cT_{\rm SE}/(4L_{\rm SE})$ is the coupling rate of the SE mode to the continuum of input modes $\hat{a}_{\rm in}$; x is the displacement of the test mass partially in reaction to the gravitational-wave tidal force $F_{\rm GW}$; the mirror motion x is coupled via the radiation-pressure force $\hat{F}_{\rm rp}$ to the cavity mode with strength $G_0 = \omega_0/L_{\rm arm}$; κ is the coupling strength due to a crystal nonlinearity under a second harmonic pump field with amplitude β and phase ϕ. The pump field is assumed to be classical and its depletion is neglected. The effect of the back-action noise can be neglected, so displacement of the mirror is coupled only to a GW strain: $x = h_0/L_{\rm arm}$.

We obtain the Langevin equations of motion for the cavity modes in the frame rotating at ω_0 and expand the quantum amplitudes into a sum of large classical amplitude and small quantum fluctuation, $\hat{a} \to A + \hat{a}$:

$$\dot{\hat{a}} = -i\omega_s\hat{a}_q + iGh_0\,, \tag{27}$$

$$\dot{\hat{a}}_q = -i\omega_s\hat{a} - \gamma\hat{a}_q + \sqrt{2\gamma}a_{\rm in} - i\chi\hat{a}_s^\dagger e^{i\phi}\,, \tag{28}$$

$$\hat{a}_{\rm out} = -\hat{a}_{\rm in} + \sqrt{2\gamma}\hat{a}_q\,, \tag{29}$$

where we defined an effective coupling strength of GW signal strain $G = \sqrt{2P_cL_{\rm arm}\omega_0/(\hbar c)}$ and optical power inside the arm cavity $P_c = \hbar\omega_0\bar{a}$, with $\bar{a}$ being an average amplitude of the mode $\hat{a}$; and the effective parametric gain $\chi = \kappa\beta$,

As we are interested in the spectral properties of the system, we transform into a Fourier domain: $\dot{\hat{a}}(t) \to -i\Omega\hat{a}(\Omega)$. The outgoing light is measured by a homodyne detector, which measures the quadratures of the light, that are defined as:

$$\hat{a}^{(1)} = \frac{\hat{a}(\Omega) + \hat{a}^\dagger(-\Omega)}{\sqrt{2}}, \qquad \hat{a}^{(2)} = \frac{\hat{a}(\Omega) - \hat{a}^\dagger(-\Omega)}{i\sqrt{2}}\,. \tag{30}$$

We obtain the input-output relations for the two quadratures by solving Eqs.(27-29):

$$\hat{a}^{(1)}_{\rm out}(\Omega) = \hat{a}^{(1)}_{\rm in}(\Omega)\frac{(\gamma-\chi)\Omega + i(\Omega^2-\omega_s^2)}{(\gamma+\chi)\Omega - i(\Omega^2-\omega_s^2)} + h_0(\Omega)\frac{2iG\sqrt{\gamma}\omega_s}{(\gamma+\chi)\Omega - i(\Omega^2-\omega_s^2)} = \tag{31}$$

$$= \hat{a}^{(1)}_{\rm in}(\Omega)\mathcal{R}_a(\Omega) + h_0(\Omega)\mathcal{T}(\Omega) \tag{32}$$

$$\hat{a}^{(2)}_{\rm out}(\Omega) = \hat{a}^{(2)}_{\rm in}(\Omega)\frac{(\gamma+\chi)\Omega + i(\Omega^2-\omega_s^2)}{(\gamma-\chi)\Omega - i(\Omega^2-\omega_s^2)}, \tag{33}$$

$$\hat{a}^{(2)}_{q}(\Omega) = \hat{a}^{(2)}_{\rm in}(\Omega)\frac{\sqrt{2\gamma}\Omega}{(\gamma+\chi)\Omega - i(\Omega^2-\omega_s^2)} + h_0(\Omega)\frac{iG\omega_s}{(\gamma+\chi)\Omega - i(\Omega^2-\omega_s^2)}, \tag{34}$$

$$\hat{a}^{(1)}(\Omega) = \hat{a}^{(2)}_{\rm in}(\Omega)\frac{i\sqrt{2\gamma}\omega_s}{(\gamma-\chi)\Omega - i(\Omega^2-\omega_s^2)}, \tag{35}$$

From these input-output relations we can obtain the sensitivity, by computing the spectral densities. We define the spectral density of the field $\hat{a}(\Omega)$ as:

$$S_a(\Omega)\delta(\Omega-\Omega') = \frac{1}{2}\left\langle \hat{a}(\Omega)\hat{a}(\Omega') + \hat{a}(\Omega')\hat{a}(\Omega)\right\rangle \ . \tag{36}$$

Then the spectral density the output noise $\hat{a}^{(c)}_{\rm out}(\Omega)$ is:

$$S_{\rm out}(\Omega) = S_{\rm in}(\Omega)|\mathcal{R}_a(\Omega)|^2\,, \tag{37}$$

where $S_{\rm in}(\Omega)$ is the spectral density of incoming light field, which we assume here to be vacuum: $S_{\rm in}(\Omega) = 1$. Assuming that we squeeze the signal quadrature of the light: $\phi = -\pi/2$, we obtain the following noise spectral density

$$S_{\rm out}(\Omega) = 1 - \frac{4\gamma\chi\Omega^2}{(\gamma+\chi)^2\Omega^2 + (\Omega^2-\omega_s^2)^2}\,, \tag{38}$$

and signal transfer function:

$$|\mathcal{T}(\Omega)|^2 = \frac{4G^2\gamma\omega_s^2}{(\gamma+\chi)^2\Omega^2 + (\Omega^2-\omega_s^2)^2}\,. \tag{39}$$

The total strain sensitivity is given by the noise normalised to the signal transfer function (subsituting the expression for the effective coupling strength G):

$$S_h(\Omega) = \frac{\hbar c}{8\omega_0 L_{\rm arm} P_c}\frac{(\gamma-\chi)^2\Omega^2 + (\Omega^2-\omega_s^2)^2}{\gamma\omega_s^2}\,, \tag{40}$$

which is the equation (9) in the Main text.

S5 Transfer matrix approach to full description

In this section we use the transfer matrix approach (*14*) to compute the sensitivity of the detector taking into account the radiation pressure noise and optical losses. We start from the same point as in the section S3, but write down the input-output relations as propagation of the field amplitudes in terms of transfer matrices for each optical element. The description is broader than strictly needed to compute the spectral density in the main text (e.g. it includes the effects of dynamical back action), but we find it helpful to use a general approach. The source code that implements these equations can be found in (*17*).

S5.1 Input-output relations

We describe a two-cavity system, as shown on Fig. S4, in terms of input and output quantum fields. Based on two-photon quadrature amplitudes we define the vector $\hat{\mathrm{a}}(\Omega) = \{\hat{a}^{(c)}(\Omega), \hat{a}^{(s)}(\Omega)\}^{\mathrm{T}}$. The signal extraction cavity can rotate the quadratures due to its detuning from resonance. The optical parametric amplification process also squeezes and rotates the quadratures. The effect of the signal recycling cavity can be described as a set of rotations and squeezing operations:

$$\hat{\mathrm{a}}_s = O(\varphi)O(\theta)\mathcal{S}O^{\dagger}(\theta)O(\phi)(\sqrt{1-\lambda_s}\hat{d}_s + \sqrt{\lambda_s}\hat{n}_1)e^{i\Omega\tau_{\mathrm{SE}}}\,, \tag{41}$$

$$\hat{\mathrm{b}}_s = -R_i\hat{\mathrm{a}}_s + T_i\hat{\mathrm{c}}\,, \tag{42}$$

$$\hat{\mathrm{c}}_s = \sqrt{1-\lambda_s}O(\phi)O(\theta)\mathcal{S}O^{\dagger}(\theta)O(\varphi)\hat{\mathrm{b}}_s e^{i\Omega\tau_{\mathrm{SE}}} + \sqrt{\lambda_s}\hat{\mathrm{n}}_2\,, \tag{43}$$

$$\hat{\mathrm{d}}_s = T_s\hat{\mathrm{a}} + R_s\hat{\mathrm{c}}_s\,, \tag{44}$$

where we denote the amplitude reflectivity and transmissivity of the signal recycling and input mirrors by $R_{s,i}, T_{s,i}$, the power loss inside the cavity (before the crystal) is λ_s; signal recycling cavity global delay $\tau_{\mathrm{SE}} = L_{\mathrm{SE}}/c$ and the phase delay due to the cavity detuning before and after

the crystal by ϕ, φ. We now introduce the squeeze angle θ and the rotation matrix

$$\forall \phi, \quad O(\phi) = \begin{bmatrix} \cos\phi & -\sin\phi \\ \sin\phi & \cos\phi \end{bmatrix}, \tag{45}$$

$$\mathcal{Y} = O(\pi/2) = \begin{bmatrix} 0 & -1 \\ 1 & 0 \end{bmatrix}, \tag{46}$$

and squeezing matrix

$$\mathcal{S} = \begin{bmatrix} e^q & 0 \\ 0 & e^{-q} \end{bmatrix}, \tag{47}$$

with q being the single-pass squeeze factor.

For the arm cavity the corresponding set of equations reads

$$\hat{\mathrm{b}} = -R_s \hat{\mathrm{a}} + T_s \hat{\mathrm{c}}_s \,, \tag{48}$$

$$\hat{\mathrm{d}} = R_i \hat{\mathrm{c}} + T_i \hat{\mathrm{a}} \,, \tag{49}$$

$$\hat{\mathrm{c}} = O(\delta_{\mathrm{arm}} \tau_{\mathrm{arm}}) \hat{\mathrm{f}} e^{i\Omega\tau_{\mathrm{arm}}} \,, \tag{50}$$

$$\hat{\mathrm{e}} = O(\delta_{\mathrm{arm}} \tau_{\mathrm{arm}}) \hat{\mathrm{d}} e^{i\Omega\tau_{\mathrm{arm}}} \,, \tag{51}$$

$$\hat{\mathrm{f}} = R_e \hat{\mathrm{e}} + T_e \hat{\mathrm{v}} + 2kR_e O(\pi/2) \mathrm{E} \hat{x}_-(\Omega) \,, \tag{52}$$

where $k = \omega/c$ is the wave vector of the main field, δ_{arm} is the arm cavity detuning and $\tau_{\mathrm{arm}} = L_{\mathrm{arm}}/c$ is the propagation time with L_{arm} being the length of the arm cavity, and c the speed of light. The field E corresponds to the classical amplitude of the field impinging on the end mirror.

We find the solution to these equations, first for the complex transmissivity and reflectivity

of the signal recycling cavity

$$
\begin{aligned}
\hat{\mathrm{b}}_s &= \mathcal{D}_b \left[-R_i T_s \mathcal{M}[\varphi,\phi]\hat{\mathrm{a}} + T_i \hat{\mathrm{c}}\right] \,, && (53)\\
\hat{\mathrm{d}}_s &= \mathcal{D}_d \left[R_s T_i \mathcal{M}[\phi,\varphi]\hat{\mathrm{c}} + T_s \hat{\mathrm{a}}\right] \,, && (54)\\
\hat{\mathrm{a}}_s &= \mathcal{M}[\varphi,\phi]\mathcal{D}_d \left[R_s T_i \mathcal{M}[\phi,\varphi]\hat{\mathrm{c}} + T_s \hat{\mathrm{a}}\right] \,, && (55)\\
\hat{\mathrm{c}}_s &= \mathcal{M}[\phi,\varphi]\mathcal{D}_b \left[-R_i T_s \mathcal{M}[\varphi,\phi]\hat{\mathrm{a}} + T_i \hat{\mathrm{c}}\right] \,, && (56)\\
& && (57)
\end{aligned}
$$

where we defined

$$
\begin{aligned}
\mathcal{M}[\phi,\psi] &= O(\phi)O(\theta)\mathcal{S}O^\dagger(\theta)O(\psi)e^{i\Omega\tau_{\rm SE}}, \forall\phi,\psi \,, && (58)\\
\mathcal{D}_b &= \left(\mathcal{I} + R_i R_s (1-\lambda_s)\mathcal{M}[\varphi,\phi]\mathcal{M}[\phi,\varphi]\right)^{-1} \,, && (59)\\
\mathcal{D}_d &= \left(\mathcal{I} + R_i R_s (1-\lambda_s)\mathcal{M}[\phi,\varphi]\mathcal{M}[\varphi,\phi]\right)^{-1} \,. && (60)
\end{aligned}
$$

That provides the input-output relations for the signal extraction cavity

$$
\begin{aligned}
\hat{\mathrm{b}} &= -\mathcal{R}_b\hat{\mathrm{a}} + \mathcal{T}_b\hat{\mathrm{c}} + \mathcal{L}_{b1}\hat{\mathrm{n}}_1 + \mathcal{L}_{b2}\hat{\mathrm{n}}_2 \,, && (61)\\
\hat{\mathrm{d}} &= \mathcal{R}_d\hat{\mathrm{c}} + \mathcal{T}_d\hat{\mathrm{a}} + \mathcal{L}_{d1}\hat{\mathrm{n}}_1 + \mathcal{L}_{d2}\hat{\mathrm{n}}_2 \,, && (62)
\end{aligned}
$$

where we introduced the transfer matrices for the fields

$$
\begin{aligned}
\mathcal{R}_b &= R_s + R_i T_s^2 (1-\lambda_s)\mathcal{M}[\phi,\varphi]\mathcal{D}_b\mathcal{M}[\varphi,\phi] \,, && (63)\\
\mathcal{R}_d &= R_i + R_s T_i^2 (1-\lambda_s)\mathcal{M}[\varphi,\phi]\mathcal{D}_d\mathcal{M}[\phi,\varphi] \,, && (64)\\
\mathcal{T}_b &= T_i T_s \sqrt{1-\lambda_s}\mathcal{M}[\phi,\varphi]\mathcal{D}_b \,, && (65)\\
\mathcal{T}_d &= T_i T_s \sqrt{1-\lambda_s}\mathcal{M}[\varphi,\phi]\mathcal{D}_d \,, && (66)\\
\mathcal{L}_{b1} &= -T_s R_i \sqrt{1-\lambda_s}\lambda_s \mathcal{M}[\varphi,\phi]\mathcal{D}_b\mathcal{M}[\phi,\varphi] \,, && (67)\\
\mathcal{L}_{b2} &= T_s R_i R_s \sqrt{\lambda_s}(1-\lambda_s)\mathcal{M}[\varphi,\phi]\mathcal{D}_b\mathcal{M}[\phi,\varphi] - \sqrt{\lambda_s} \,, && (68)\\
\mathcal{L}_{d1} &= -T_i R_i R_s \sqrt{\lambda_s}(1-\lambda_s)\mathcal{M}[\phi,\varphi]\mathcal{D}_d\mathcal{M}[\varphi,\phi] + \sqrt{\lambda_s} \,, && (69)\\
\mathcal{L}_{d2} &= T_i R_s \sqrt{\lambda_s(1-\lambda_s)}\mathcal{M}[\phi,\varphi]\mathcal{D}_d \,. && (70)
\end{aligned}
$$

Now we can derive the fields for the arm cavity yielding

$$
\begin{aligned}
\hat{\mathrm{c}} &= R_e\mathcal{D}_c O(\delta_{\mathrm{arm}}\tau_{\mathrm{arm}})^2\mathcal{T}_d\hat{\mathrm{a}}e^{2i\Omega\tau_{\mathrm{arm}}} + T_e\mathcal{D}_c O(\delta_{\mathrm{arm}}\tau_{\mathrm{arm}})\hat{\mathrm{v}}e^{i\Omega\tau_{\mathrm{arm}}} + \\
&+ R_e\mathcal{D}_c O(\delta_{\mathrm{arm}}\tau_{\mathrm{arm}})^2\left(\mathcal{L}_{d1}\hat{\mathrm{n}}_1 + \mathcal{L}_{d2}\hat{\mathrm{n}}_2\right)e^{2i\Omega\tau_{\mathrm{arm}}} + \\
&+ 2kR_e\mathcal{D}_c O(\delta_{\mathrm{arm}}\tau_{\mathrm{arm}})\mathcal{Y}\mathrm{E}\hat{x}_-(\Omega)e^{i\Omega\tau_{\mathrm{arm}}} \qquad (71)\\
\hat{\mathrm{e}} &= \mathcal{D}_e O(\delta_{\mathrm{arm}}\tau_{\mathrm{arm}})\mathcal{T}_d\hat{\mathrm{a}}e^{i\Omega\tau_{\mathrm{arm}}} + T_e\mathcal{D}_e O(\delta_{\mathrm{arm}}\tau_{\mathrm{arm}})\mathcal{R}_d O(\delta_{\mathrm{arm}}\tau_{\mathrm{arm}})\hat{\mathrm{v}}e^{2i\Omega\tau_{\mathrm{arm}}} + \\
&+ \mathcal{D}_e O(\delta_{\mathrm{arm}}\tau_{\mathrm{arm}})\left(\mathcal{L}_{d1}\hat{\mathrm{n}}_1 + \mathcal{L}_{d2}\hat{\mathrm{n}}_2\right)e^{i\Omega\tau_{\mathrm{arm}}} + \\
&+ 2kR_e\mathcal{D}_e O(\delta_{\mathrm{arm}}\tau_{\mathrm{arm}})\mathcal{R}_d O(\delta_{\mathrm{arm}}\tau_{\mathrm{arm}})\mathcal{Y}\mathrm{E}\hat{x}_-(\Omega)e^{2i\Omega\tau_{\mathrm{arm}}}\,, \qquad (72)
\end{aligned}
$$

where

$$
\begin{aligned}
\mathcal{D}_c &= \left(\mathcal{I} - R_e O(\delta_{\mathrm{arm}}\tau_{\mathrm{arm}})^2\mathcal{R}_d e^{2i\Omega\tau_{\mathrm{arm}}}\right)^{-1}\,, \qquad (73)\\
\mathcal{D}_e &= \left(\mathcal{I} - R_e O(\delta_{\mathrm{arm}}\tau_{\mathrm{arm}})\mathcal{R}_d O(\delta_{\mathrm{arm}}\tau_{\mathrm{arm}})e^{2i\Omega\tau_{\mathrm{arm}}}\right)^{-1}\,. \qquad (74)
\end{aligned}
$$

Finally, we find the outgoing field to be

$$
\hat{\mathrm{b}} = -\mathcal{R}\hat{\mathrm{a}} + \mathcal{T}\hat{\mathrm{v}} + \mathcal{Z}\hat{x}_-(\Omega) + \mathcal{L}_1\hat{\mathrm{n}}_1 + \mathcal{L}_2\hat{\mathrm{n}}_2\,, \qquad (75)
$$

where we defined the transfer matrices:

$$
\begin{aligned}
\mathcal{R} &= \mathcal{R}_b - R_e\mathcal{T}_b\mathcal{D}_c O(\delta_{\mathrm{arm}}\tau_{\mathrm{arm}})^2\mathcal{T}_d e^{2i\Omega\tau_{\mathrm{arm}}}\,, \qquad (76)\\
\mathcal{T} &= T_e\mathcal{T}_b\mathcal{D}_c O(\delta_{\mathrm{arm}}\tau_{\mathrm{arm}})e^{i\Omega\tau_{\mathrm{arm}}}\,, \qquad (77)\\
\mathcal{Z} &= 2kR_e\mathcal{T}_b\mathcal{D}_c O(\delta_{\mathrm{arm}}\tau_{\mathrm{arm}})\mathcal{Y}\mathrm{E}e^{i\Omega\tau_{\mathrm{arm}}}\,, \qquad (78)\\
\mathcal{L}_1 &= R_e\mathcal{T}_b\mathcal{D}_c O(\delta_{\mathrm{arm}}\tau_{\mathrm{arm}})O(\delta_{\mathrm{arm}}\tau_{\mathrm{arm}})\mathcal{L}_{d1}e^{2i\Omega\tau_{\mathrm{arm}}} + \mathcal{L}_{b1}\,, \qquad (79)\\
\mathcal{L}_2 &= R_e\mathcal{T}_b\mathcal{D}_c O(\delta_{\mathrm{arm}}\tau_{\mathrm{arm}})O(\delta_{\mathrm{arm}}\tau_{\mathrm{arm}})\mathcal{L}_{d2}e^{2i\Omega\tau_{\mathrm{arm}}} + \mathcal{L}_{b2}\,. \qquad (80)
\end{aligned}
$$

(81)

S5.2 Radiation pressure

The radiation pressure force acting on the mirrors has three contributions. First, there is a constant force due to the classical high-power optical field. It induces a constant shift of the mirror, which can be compensated with classical feedback. Second, there is a dynamical classical part, which is amplified by opto-mechanical parametric amplification and which belongs to the optical spring, and third a fluctuating force due to the uncertainty in the amplitude quadrature of the light. The latter corresponds to the quantum back-action force of the carrier light. Following (5), we assume the input test mass to be fixed, and twice the back action imposed on the back mirror instead (which leads to introduction of effective light power). Such approximation is valid when the transmission of front mirror is small, such that the amplitudes of the fields acting on the front and back mirrors are almost equal (which is the case in our consideration).

$$F^{ba} = \hbar k(\mathrm{E}^\dagger \hat{\mathrm{e}}(\Omega) + \mathrm{F}^\dagger \hat{\mathrm{f}}(\Omega)) = F_{fl}(\Omega) - \mathcal{K}(\Omega) x_-(\Omega)\,, \tag{82}$$

where we split the back-action into the noise part $F_{fl}(\Omega)$ and position-dependent optical spring force with spring constant $\mathcal{K}(\Omega)$. Taking into account that $\mathrm{F} = R_e\mathrm{E}$, we find the equations for these contributions:

$$\begin{aligned}
F^{fl}(\Omega) &= \hbar k(1+R_e^2)\mathrm{E}^\dagger \mathcal{D}_e O(\delta_{\mathrm{arm}}\tau_{\mathrm{arm}}) e^{i\Omega\tau_{\mathrm{arm}}} (\mathcal{T}_d \hat{\mathrm{a}} + \mathcal{L}_{d1}\hat{\mathrm{n}}_1 + \mathcal{L}_{d2}\hat{\mathrm{n}}_2) + \\
&+ \hbar k T_e \mathrm{E}^\dagger \mathcal{L}_v \hat{\mathrm{v}}\,, &(83)\\
\mathcal{L}_v &= (1+R_e^2)\mathcal{D}_e O(\delta_{\mathrm{arm}}\tau_{\mathrm{arm}})\mathcal{R}_d O(\delta_{\mathrm{arm}}\tau_{\mathrm{arm}}) e^{i\Omega\tau_{\mathrm{arm}}} + R_e\,, &(84)\\
\mathcal{K}(\Omega) &= -2\hbar k^2(1+R_e^2)R_e \mathrm{E}^\dagger \mathcal{D}_e O(\delta_{\mathrm{arm}}\tau_{\mathrm{arm}})\mathcal{R}_d O(\delta_{\mathrm{arm}}\tau_{\mathrm{arm}})\mathcal{Y}\mathrm{E} e^{2i\Omega\tau_{\mathrm{arm}}} - \\
&- 2\hbar k^2 R_e^2 \mathrm{E}^\dagger \mathcal{Y}\mathrm{E}\,. &(85)
\end{aligned}$$

Without loss of generality we choose the phase of the classical amplitude such that:

$$\mathrm{E} = \sqrt{2}E\{1, 0\}^{\mathrm{T}}\,, \tag{86}$$

where the amplitude E is connected to the power in the cavity as $P_c = 2P_{\mathrm{arm}} = \hbar\omega_p|E|^2$, where P_{arm} is a power in the corresponding Michelson interferometer (5).

The equation of motion for the test mass taking into account the radiation pressure force:

$$\hat{x}_-(\Omega) = \chi(\Omega)\left[F^{fl}(\Omega) - \mathcal{K}(\Omega)x_-(\Omega)\right]\,, \tag{87}$$

where $\chi(\Omega) = (-m\Omega^2)^{-1}$ is the mechanical suspeptibility for the free mass m. This allows us to introduce an effective susceptibility:

$$\chi_{\mathrm{eff}}(\Omega) = (\chi^{-1} + \mathcal{K}(\Omega))^{-1}\,, \tag{88}$$

such that $x_-(\Omega) = \chi_{\mathrm{eff}}(\Omega)F^{fl}(\Omega)$.

S5.3 Detection

The presence of optical loss in the readout path, including the detection loss, leads to a loss of quantum correlations due to mixing with vacuum. We model this loss with a beam splitter of power transmissivity $\eta = 1 - \lambda_r$ and reflectivity (loss) $1 - \eta = \lambda_r$ which mixes in vacuum n:

$$\tilde{\mathrm{b}}(\Omega) = \sqrt{\eta}\mathrm{b}(\Omega) + \sqrt{1-\eta}\mathrm{n}\,. \tag{89}$$

The balanced homodyne detection on the output $\tilde{\mathrm{b}}$ at homodyne angle ζ provides the values

$$y(\Omega) = \{\cos\zeta, \sin\zeta\}^{\mathrm{T}}\tilde{\mathrm{b}}(\Omega) = \mathcal{H}^{\mathrm{T}}\tilde{\mathrm{b}}(\Omega) = \sqrt{\eta}\mathcal{H}^{\mathrm{T}}\left(-\mathcal{R}\hat{\mathrm{a}} + \mathcal{T}\hat{\mathrm{v}} + \mathcal{L}_{b1}\hat{\mathrm{n}}_1 + \mathcal{L}_{b2}\hat{\mathrm{n}}_2\right) + \sqrt{\eta}\mathcal{H}^{\mathrm{T}}\mathcal{Z}\hat{x}_-(\Omega) + \sqrt{1-\eta}\mathcal{H}^{\mathrm{T}}\mathrm{n}(\Omega), \tag{90}$$

which we renormalize to the differential mirror displacement

$$\tilde{y} = \frac{\mathcal{H}^{\mathrm{T}}\left(-\mathcal{R}\hat{\mathrm{a}} + \mathcal{T}\hat{\mathrm{v}} + \mathcal{L}_{b1}\hat{\mathrm{n}}_1 + \mathcal{L}_{b2}\hat{\mathrm{n}}_2\right)}{\mathcal{H}^{\mathrm{T}}\mathcal{Z}} + \frac{\sqrt{1-\eta}\mathcal{H}^{\mathrm{T}}\mathrm{n}}{\sqrt{\eta}\mathcal{H}^{\mathrm{T}}\mathcal{Z}} + \hat{x}_-(\Omega)\,. \tag{91}$$

We implement the injection of the squeezing from the outside, by defining an action of the squeezing operation on the input field $\hat{\mathrm{a}}$ as:

$$\hat{\mathrm{a}} = \mathcal{S}_{\mathrm{ext}}[\phi_{\mathrm{ext}}]\hat{\mathrm{a}}^{\mathrm{vac}}, \tag{92}$$

where $\hat{\mathrm{a}}^{\mathrm{vac}}$ is the vacuum field before squeezing, and the squeezing matrix with squeeze factor q_{ext} and squeeze angle ϕ_{ext} is defined as

$$\mathcal{S}_{\mathrm{ext}} = O(\phi_{\mathrm{ext}}) \begin{bmatrix} e^{q_{\mathrm{ext}}} & 0 \\ 0 & e^{-q_{\mathrm{ext}}} \end{bmatrix} O(-\phi_{\mathrm{ext}}). \tag{93}$$

All other fields $\hat{\mathrm{v}}, \hat{\mathrm{n}}, \hat{\mathrm{n}}_1, \hat{\mathrm{n}}_1$ are in the vacuum state.

From this we get the spectral density for this output

$$S_x(\Omega) = S_{xx}(\Omega) + 2\mathrm{Re}[\chi^*_{\mathrm{eff}}(\Omega) S_{xF}(\Omega)] + |\chi_{\mathrm{eff}}(\Omega)|^2 S_{FF}(\Omega)\,, \tag{94}$$

where

$$S_{xx} = \frac{\mathcal{H}^{\mathrm{T}}(\mathcal{R}\mathcal{S}_{\mathrm{ext}}\mathcal{S}^\dagger_{\mathrm{ext}}\mathcal{R}^\dagger + \mathcal{T}\mathcal{T}^\dagger + \mathcal{L}_{b1}\mathcal{L}^\dagger_{b1} + \mathcal{L}_{b2}\mathcal{L}^\dagger_{b2})\mathcal{H}}{|\mathcal{H}^{\mathrm{T}}\mathcal{Z}|^2} + \frac{1-\eta}{\eta}\frac{1}{|\mathcal{H}^{\mathrm{T}}\mathcal{Z}|^2}\,, \tag{95}$$

$$\begin{aligned} S_{FF} &= \hbar^2 k^2 (1+R_e^2)^2 \mathrm{E}^\dagger \mathcal{D}_e O(\delta_{\mathrm{arm}}\tau_{\mathrm{arm}}) \left(\mathcal{T}_d \mathcal{S}_{\mathrm{ext}}\mathcal{S}^\dagger_{\mathrm{ext}}\mathcal{T}^\dagger_d + \mathcal{L}_{d1}\mathcal{L}^\dagger_{d1} + \mathcal{L}_{d2}\mathcal{L}^\dagger_{d2}\right) O^\dagger(\delta_{\mathrm{arm}}\tau_{\mathrm{arm}})\mathcal{D}^\dagger_e \mathrm{E} + \\ &+ \quad \hbar^2 k^2 T_e^2 \mathrm{E}^\dagger \mathcal{L}_v \mathcal{L}^\dagger_v \mathrm{E}\,, \end{aligned} \tag{96}$$

$$\begin{aligned} S_{xF} &= \frac{\hbar k}{\mathcal{H}^{\mathrm{T}}\mathcal{Z}} \Big((1+R_e^2)\mathcal{H}^{\mathrm{T}}(-\mathcal{R}\mathcal{S}_{\mathrm{ext}}\mathcal{S}^\dagger_{\mathrm{ext}}\mathcal{T}^\dagger_d + \mathcal{L}_{b1}\mathcal{L}^\dagger_{d1} + \mathcal{L}_{b2}\mathcal{L}^\dagger_{d2})O^\dagger(\delta_{\mathrm{arm}}\tau_{\mathrm{arm}})\mathcal{D}^\dagger_e \mathrm{E} e^{-i\Omega\tau_{\mathrm{arm}}} + \\ &+ \quad T_e \mathcal{H}^{\mathrm{T}}\mathcal{T}\mathcal{L}^\dagger_v \mathrm{E}\Big)\,. \end{aligned} \tag{97}$$

Finally we normalize the spectral density to the gravitational-wave strain yielding (taking into account the effects of high-frequency corrections (*18*))

$$S_h(\Omega) = S_x(\Omega)\frac{4}{m^2 L^2 \Omega^4 |\chi_{\mathrm{eff}}(\Omega)|^2}\frac{\Omega^2\tau^2_{\mathrm{arm}}}{\sin^2\Omega\tau_{\mathrm{arm}}}\,. \tag{98}$$

S5.4 Filter cavities

Filter cavities on the can be used to create a necessary frequency dependence of quantum correlations, such that the QRPN is suppressed or evaded completely. There are two scenarios, input filter cavity, where the injected squeezing becomes frequency dependent, and output filter cavity, where the homodyne detection becomes frequency dependent. We follow (*14*) and consider a lossless filter cavity, so that the only effect of the cavity is a frequency-dependent rotation of the input squeezed state $\hat{\mathrm{a}} \to O[\theta_f(\Omega)]\hat{\mathrm{a}}$ or output $\mathrm{b}(\Omega) \to O[\theta_f(\Omega)]\mathrm{b}(\Omega)$, by the angle

$$\theta_f(\Omega) = \arctan \frac{2\gamma_f \delta_f}{\gamma_f^2 - \delta_f^2 + \Omega^2}\,, \tag{99}$$

where γ_f is the filter cavity bandwidth, and δ_f is it's detuning from resonance. To obtain the spectral corresponding spectral densities it's sufficient to modify the squeeze angle $\phi_{\text{ext}} \to \phi_{\text{ext}} + \theta_f(\Omega)$ or homodyne angle $\zeta \to \zeta - \theta_f(\Omega)$ in the equations for the spectral density Eq. 94. The optimal detuning is on the slope of the cavity resonance $\delta_f = \gamma_f$, and the exact choice of cavity linewidth depends on the parameters of the detector, including the internal squeezing strength and readout loss.

References

1. C. M. Caves, Quantum-mechanical noise in an interferometer, *Physical Review D* **23**, 1693 (1981).

2. E. Knyazev, K. Y. Spasibko, M. V. Chekhova, F. Y. Khalili, Quantum tomography enhanced through parametric amplification, *New Journal of Physics* **20**, 013005 (2018).

3. E. Schreiber, Gravitational-wave detection beyond the quantum shot-noise limit, Ph.D. thesis, Leibniz Universität Hannover (2017).

4. J. Steinlechner, *et al.*, Absorption Measurements of Periodically Poled Potassium Titanyl Phosphate (PPKTP) at 775 nm and 1550 nm, *Sensors* **13**, 565 (2013).

5. A. Buonanno, Y. Chen, Scaling law in signal recycled laser-interferometer gravitational-wave detectors, *Physical Review D* pp. 1–23 (2003).

6. LIGO Scientific Collaboration and Virgo Collaboration, Advanced LIGO, *Classical and Quantum Gravity* **32**, 074001 (2015).

7. H. Vahlbruch, S. Chelkowski, K. Danzmann, R. Schnabel, Quantum engineering of squeezed states for quantum communication and metrology, *New Journal of Physics* **9**, 371 (2007).

8. H. Vahlbruch, M. Mehmet, K. Danzmann, R. Schnabel, Detection of 15 dB Squeezed States of Light and their Application for the Absolute Calibration of Photoelectric Quantum Efficiency, *Physical Review Letters* **117**, 1 (2016).

9. M. Korobko, F. Y. Khalili, R. Schnabel, Engineering the optical spring via intra-cavity optical-parametric amplification, *Physics Letters, Section A: General, Atomic and Solid State Physics* **382**, 2238 (2018).

10. H. Yang, *et al.*, Gravitational wave spectroscopy of binary neutron star merger remnants with mode stacking, *Phys. Rev.* **D97**, 024049 (2018).

11. H. Miao, H. Yang, D. Martynov, Towards the design of gravitational-wave detectors for probing neutron-star physics, *Physical Review D* **98**, 044044 (2018).

12. A. Bauswein, N. Stergioulas, H.-T. Janka, Exploring properties of high-density matter through remnants of neutron-star mergers, *The European Physical Journal A* **52**, 56 (2016).

13. H. Shen, H. Toki, K. Oyamatsu, K. Sumiyoshi, Relativistic equation of state of nuclear matter for supernova and neutron star, *Nuclear Physics A* **637**, 435 (1998).

14. S. L. Danilishin, F. Y. Khalili, Quantum Measurement Theory in Gravitational-Wave Detectors, *Living Reviews in Relativity* **15** (2012).

15. C. M. Caves, B. L. Schumaker, New formalism for two-photon quantum optics. I. Quadrature phases and squeezed states, *Physical Review A* **31**, 3068 (1985).

16. B. L. Schumaker, C. M. Caves, New formalism for two-photon quantum optics. II. Mathematical foundation and compact notation, *Physical Review A* **31**, 3093 (1985).

17. M. Korobko, Supplementary code, doi:10.5281/zenodo.3597327 (2020).

18. M. Rakhmanov, J. D. Romano, J. T. Whelan, High-frequency corrections to the detector response and their effect on searches for gravitational waves, *Classical and Quantum Gravity* **25** (2008).
